# Assessment of the stoichiometry and efficiency of CO_2_ fixation coupled to reduced sulfur oxidation

**DOI:** 10.3389/fmicb.2015.00484

**Published:** 2015-05-21

**Authors:** Judith M. Klatt, Lubos Polerecky

**Affiliations:** ^1^Max Planck Institute for Marine MicrobiologyBremen, Germany; ^2^Department of Earth Sciences – Geochemistry, Faculty of Geosciences, Utrecht UniversityUtrecht, Netherlands

**Keywords:** sulfur oxidation pathways, chemolithoautotrophy, energy conservation efficiency, stoichiometry, sulfur cycle, carbon cycle

## Abstract

Chemolithoautotrophic sulfur oxidizing bacteria (SOB) couple the oxidation of reduced sulfur compounds to the production of biomass. Their role in the cycling of carbon, sulfur, oxygen, and nitrogen is, however, difficult to quantify due to the complexity of sulfur oxidation pathways. We describe a generic theoretical framework for linking the stoichiometry and energy conservation efficiency of autotrophic sulfur oxidation while accounting for the partitioning of the reduced sulfur pool between the energy generating and energy conserving steps as well as between the main possible products (sulfate vs. zero-valent sulfur). Using this framework, we show that the energy conservation efficiency varies widely among SOB with no apparent relationship to their phylogeny. Aerobic SOB equipped with reverse dissimilatory sulfite reductase tend to have higher efficiency than those relying on the complete Sox pathway, whereas for anaerobic SOB the presence of membrane-bound, as opposed to periplasmic, nitrate reductase systems appears to be linked to higher efficiency. We employ the framework to also show how limited rate measurements can be used to estimate the primary productivity of SOB without the knowledge of the sulfate-to-zero-valent-sulfur production ratio. Finally, we discuss how the framework can help researchers gain new insights into the activity of SOB and their niches.

## Introduction

Autotrophic sulfur oxidizing bacteria (SOB) comprise a phylogenetically diverse group of microbes that obtain the energy required for growth from the oxidation of reduced sulfur compounds. Prominent natural habitats of SOB are hydrothermal vents, where these bacteria live in symbiotic association with invertebrates or, when free-living, form microbial mats (e.g., Sievert and Vetriani, 2012). SOB are found also on top of organic-rich marine sediments, where they typically form conspicuous and often quite extensive mats. For instance, on the continental shelf off the Namibian coast, such mats cover an area comparable to the size of Austria (>80,000 km^2^; Brüchert et al., 2006). In addition to natural habitats, SOB are important also in industrial applications, where they are used, for instance, for waste water treatment by biodesulfurisation (Janssen et al., 2009).

A common feature of environments inhabited by SOB is the encounter of sulfide or other reduced sulfur species with a terminal electron acceptor (TEA) such as oxygen or nitrate. This encounter creates a chemical disequilibrium from which energy can be harvested and conserved in the form of biomass. While the general validity of this concept is well established, there are still significant gaps in our understanding of systems driven by chemical energy derived from reduced sulfur oxidation. This is because the reactions performed by autotrophic SOB are highly complex and the assessment of the stoichiometry of sulfur oxidation is not trivial, even for cultivated SOB.

In chemolithoautotrophic SOB known to date the energy gained from the reduced sulfur oxidation drives reverse electron transport within the membrane, which generates sufficiently negative electron potential to reduce an electron carrier (e.g., NAD^+^) with the reduced sulfur compound as the electron donor (e.g., Harold, 1986). Thus, the reduced sulfur compound is used as the electron donor for the reduction of both CO_2_ (via the electron carrier) and TEA. The partitioning of the reduced sulfur pool between these two reductive processes, which is related to the efficiency of energy conservation and the growth rate per substrate utilized, is poorly understood and varies substantially among the different cultivated SOB (Kelly, 1999). Additionally, the end product of sulfur oxidation (e.g., sulfate vs. zero-valent sulfur) is also variable and appears to exhibit a degree of flexibility even within individual organisms, as shown for several *Beggiatoa* strains (Nelson et al., 1986; Hagen and Nelson, 1997; Berg et al., 2014).

**Figure 1:**
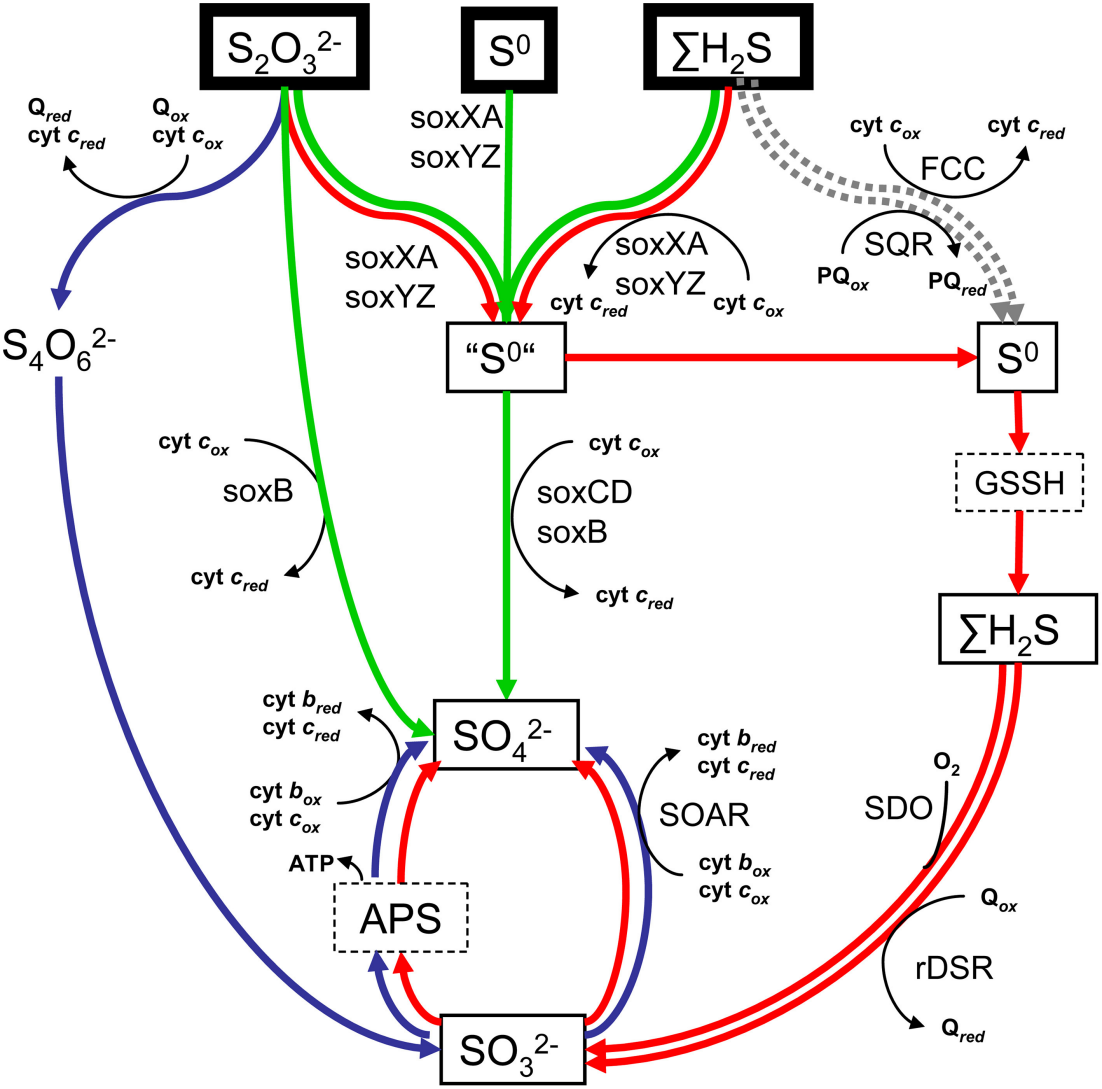
**Scheme of the main pathways of reduced sulfur oxidation**. Green arrows indicate the traditional Sox pathway, blue arrows the tetrathionate (SI_4_) pathway and red arrows correspond to the branched pathway for thiosulfate oxidation. Possible entry sites of H_2_S that are not part of these traditional pathways are shown with a gray dotted arrow. FCC = flavocytochrome c:oxidoreductase; SQR = sulfide:quinine:oxidoreductase; GSSH = S-sulfanylglutathione; APS = adenosine-5′-phosphosulfate; SOAR = sulfite:cyt c:oxidoreductase; rDSR = reverse dissimilatory sulfate reductase; SDO = sulfur dioxygenase; soxXA, soxYZ, and soxB are subunits of the thiosulfate-oxidizing multi-enzyme (TOMES) complex.

This complexity is echoed in the diversity of pathways by which SOB oxidize the reduced sulfur compound. As summarized in Figure 1, three main pathways have been identified so far (reviewed, e.g., by Ghosh and Dam, 2009): (i) the Sox pathway mediated by the thiosulfate-oxidizing multi-enzyme (TOMES) complex, (ii) the tetrathionate (SI_4_) pathway of thiosulfate oxidation, and (iii) the rather recently described “branched” pathway. The occurrence of individual enzymes of the traditional pathways does not appear to be linked to the phylogenetic identity of SOB. This phenomenon is generally explained by horizontal gene transfer, which is possibly also responsible for the co-occurrence and linkage of specific enzymes or even several complete pathways in the same organism (e.g., Ghosh and Dam, 2009). In addition to the diverse sulfur oxidation pathways, the complexity of autotrophic sulfur oxidation by SOB is further increased by the fact that they can employ different terminal oxidases for TEA reduction and two possible pathways for CO_2_ fixation (namely the Calvin-Benson-Bassham cycle, hereafter referred to as the Calvin cycle, or the reverse tricarboxylic acid (rTCA) cycle; e.g., Hügler and Sievert, 2011; Sievert and Vetriani, 2012).

The complexity of SOB physiology and sulfur oxidation biochemistry hampers our ability to quantify the contribution of SOB to sulfur, carbon, nitrogen, and oxygen cycling in a given environment, as well as to understand their niches. This is especially true for environments with fluctuating conditions, where also the products and intermediates of sulfur oxidation vary. Additionally, this complexity hinders the development of industrial applications that use SOB for processes such as waste water biodesulfurisation, which require precise control and predictability of SOB activity.

In this study we describe a generic theoretical framework for a rapid quantitative assessment of chemolithoautotrophic sulfur oxidation. First, we demonstrate how to use it for the quantification of the stoichiometry and energy conservation efficiency of autotrophic sulfur oxidation from rate measurements of the reactants involved. Second, we apply it on literature data and identify possible links between the energy conservation efficiency and the different oxidative and reductive pathways employed by SOB. Third, we suggest how it can be used to estimate chemolithoautotrophic primary productivity by SOB from limited rate measurements. Finally, we discuss how our framework makes it possible to formally relate the capabilities of SOB (e.g., stoichiometry and efficiency) to their environment and thus gain insight into the differentiation of their niches.

## Materials and methods

### Generalized equations for aerobic sulfide oxidation

The generalized mass-balanced equations for aerobic sulfide oxidation coupled to CO_2_ fixation are derived by considering the energy generating and energy conserving steps separately. The energy generating step is performed with zero-valent sulfur (S^0^) and sulfate (SO^2−^_4_) as two possible end products. The corresponding reactions are written as (Nelson et al., 1986; Kelly, 1999)

(1) $$\;{\text{H}}_{2}\text{S}+0.5\;{\text{O}}_{2}\to {\text{S}}^{0}+{\text{H}}_{2}\text{O},$$

(2) $${\text{H}}_{2}\text{S}+2\;{\text{O}}_{2}\to {\text{SO}}_{4}^{2-}{+2\text{H}}^{+}.$$

When both reactions occur simultaneously, the generalized equation for the energy generating step is

(3) $$\begin{array}{l} {\text{H}}_{2}\text{S}+(2-1.5x)\;{\text{O}}_{2}\to \\ x\;{\text{S}}^{0}+(1-x)\;{\text{SO}}_{4}^{2-}+x\;{\text{H}}_{2}\text{O}+\;(2-2x)\;{\text{H}}^{+}, \end{array}$$

where *x* and (1–*x*) are the parts of the total H_2_S pool oxidized to S^0^ and SO^2−^_4_, respectively (0≤x≤1).

In SOB, H_2_S serves not only as the energy source but also as the electron donor for the reduction of CO_2_. Analogously to the energy generating step, this can occur with S^0^ or SO^2−^_4_ as two possible end products, i.e.,

(4) $$\;{\text{H}}_{2}\text{S }+\;0.5\;{\text{CO}}_{2}\to {\text{S}}^{0}\;+\;0.5{\text{CH}}_{2}\text{O }+\;0.5{\text{H}}_{2}\text{O},$$

(5) $${\text{H}}_{2}\text{S }+\;2\;{\text{CO}}_{2}\;+\;2\;{\text{H}}_{2}\text{O}\to {\text{SO}}_{4}^{2-}\;+\;{2\text{CH}}_{2}\text{O }+\;{2\text{H}}^{+}.$$

A critical assumption in our derivation is that the S^0^:SO^2−^_4_ product ratio (defined by *x*) is the same for both the energy generating and CO_2_ fixing steps. Consequently, the generalized equation for the CO_2_ fixing step is written as

(6) $$\begin{array}{l} {\text{H}}_{2}\text{S }+\;(2-1.5x){\text{CO}}_{2}\;+\;(2-2.5x){\text{H}}_{2}\text{O}\to \\ x{\text{S}}^{0}+\;(1-x){\text{SO}}_{4}^{2-}\;+\;(2-1.5x){\text{CH}}_{2}\text{O }+\;(2-2x){\text{H}}^{+}. \end{array}$$

To arrive at a generalized equation for the complete aerobic sulfide oxidation coupled to CO_2_ reduction, we assume that part *y* (0≤y≤1) of the total H_2_S pool is used for energy generation (Equation 3) while the remaining part 1–*y* is used for CO_2_ reduction (Equation 6). Thus, by summing Equation (3) multiplied with *y* and Equation (6) multiplied with 1–*y* we obtain

(7) $$\begin{array}{l} {\text{H}}_{2}\text{S }+\;\nu_{O2}\;{\text{O}}_{2}\;+\;\nu_{CO2}\;{\text{CO}}_{2}\to \\ \nu_{S0}\;{\text{S}}^{0}\;+\;\nu_{SO4}\;{\text{SO}}_{4}^{2-}+\;\nu_{orgC}\;{\text{CH}}_{2}\text{O }+\;\nu_{H2O}{\text{H}}_{2}\text{O}\\ +\;\nu_{H+}\;{\text{H}}^{+}, \end{array}$$

where the stoichiometric coefficients of the reactants involved are ν_O2_ = y(2−1.5x), ν_CO2_ = ν_orgC_ = (1−y)(2−1.5x), ν_S0_ = x, ν_SO4_ = (1−x), ν_H2O_ = yx−(1−y)(2−2.5x) and ν_H+_ = 2(1−x). Equation (7) is the general mass-balanced equation for aerobic sulfide oxidation coupled to CO_2_ fixation.

### Efficiency of energy conservation: the traditional calculation approach

The thermodynamic efficiency of energy conservation, ε, is generally defined as the ratio between the Gibbs free energies of the endergonic (energy-consuming) and exergonic (energy-generating) reactions. Specifically for aerobic sulfide oxidation, where CO_2_ reduction (Equation 6 multiplied by 1−*y*) and O_2_ reduction (Equation 3 multiplied by *y*) are the endergonic and exergonic reactions, respectively, ε is written as

(8) $$\varepsilon_{\text{I}}=\frac{\left(1-y)\Delta G_{r}({\text{CO}}_{2}\text{ red}\right)}{-y\Delta G_{r}({\text{O}}_{2}\text{ red})},$$

where Δ**G_r_** is expressed per mole of H_2_S oxidized. At non-standard conditions, the Gibbs free energy of a reaction is calculated as

(9) $$\Delta G_{r}=\Delta G_{r}^{0}+\text{RT ln }Q,$$

where Δ*G*^0^_*r*_ is Δ*G_r_* at standard biochemical conditions (pH = 7, reactant concentrations 1 M, temperature 25°C), R is the universal gas constant, T is temperature, and *Q* is the ratio of the activity coefficients of the products and substrates involved, which can be approximated by the ratio of the corresponding concentrations (Thauer et al., 1977). Using Equations (3) and (6), the respective ΔG_*r*_ values in Equation (8) are calculated as

(10) $$\begin{array}{l} \Delta G_{r}({\text{O}}_{2}\text{red})=\text{​​​​}x\Delta G_{f}^{0}({\text{S}}^{0})+(1-x)\Delta G_{f}^{0}({\text{SO}}_{4}^{2-})\\ \;+\text{​​​​}x\Delta G_{f}^{0}({\text{H}}_{2}\text{O})-\Delta G_{f}^{0}({\text{H}}_{2}\text{S})\\ \;-\text{​​​​}(2-1.5x)\Delta G_{f}^{0}({\text{O}}_{2})+\text{RT ln }Q_{1}, \end{array}$$

(11) $$\begin{array}{l} \Delta G_{r}({\text{CO}}_{2}\text{red})=\text{​​​​}x\Delta G_{f}^{0}({\text{S}}^{0})+(1-x)\Delta G_{f}^{0}({\text{SO}}_{4}^{2-})\\ \;-\text{​​​​}\Delta G_{f}^{0}({\text{H}}_{2}\text{S})-(2-2.5x)\Delta G_{f}^{0}({\text{H}}_{2}\text{O})\\ \;+\text{​​​​}(2-1.5x)[\Delta G_{f}^{0}({\text{CH}}_{2}\text{O})-\Delta G_{f}^{0}({\text{CO}}_{2})]\\ \;+\text{​​​​RT ln}Q_{2}, \end{array}$$

where the Gibbs free energies of formation of the respective reactants at standard biochemical conditions, Δ*G*^0^_*f*_, are tabulated in Thauer et al. (1977) and the quotients *Q*_1_ and *Q*_2_ are given by

(12) $$Q_{1}=\frac{{\left[{\text{SO}}_{4}^{2-}\right]}^{\left(1-x\right)}\;{\left[{\text{H}}^{+}\right]}^{\left(2-2x\right)}}{[{\text{H}}_{2}\text{S}]\;{\left[{\text{O}}_{2}\right]}^{\left(2-1.5x\right)}},$$

(13) $$Q_{2}=\frac{{\left[{\text{SO}}_{4}^{2-}\right]}^{\left(1-x\right)}\;{\left[{\text{H}}^{+}\right]}^{\left(2-2x\right)}}{[{\text{H}}_{2}\text{S}]\;{\left[{\text{CO}}_{2}\right]}^{\left(2-1.5x\right)}}.$$

Equations (8–13) summarize the traditional approach for the calculations of the energy conservation efficiency of autotrophic aerobic sulfide oxidation (e.g., Nelson and Hagen, 1995).

### Factorization of the traditional energy conservation efficiency

The general aim of calculating the efficiency of energy conservation is to gain insights into how the energy generated by an exergonic reaction is converted into the biochemical currency ATP and how this ATP is further utilized to drive endergonic reactions. For the specific case of autotrophic sulfur oxidation, we divide the flow of energy into three steps: conversion of the Gibbs free energy released by sulfur oxidation into ATP, transfer of this ATP to the site of CO_2_ reduction (that is, not for processes associated with “cell maintenance”), and ATP utilization for driving CO_2_ reduction (see Figure S1). The efficiency of the first step, ε_SO_, characterizes the efficiency of the sulfur oxidation pathway. It is calculated as

(14) $$\varepsilon_{\text{SO}}=\frac{\Delta E_{r}({\text{O}}_{2}\text{ red})}{\Delta G_{r}({\text{O}}_{2}\text{ red})},$$

where Δ*E_r_*(O_2_ red) describes the energy gained, in the form of ATP, from the reduction of O_2_. Analogously, the efficiency of ATP utilization for the reduction of CO_2_, ε_u_, is calculated as

(15) $$\varepsilon_{\text{u}}=\frac{\Delta G_{r}({\text{CO}}_{2}\text{ red})}{\Delta E_{r}({\text{CO}}_{2}\text{ red})},$$

where Δ*E_r_*(CO_2_ red) describes the energy requirements, in the form of ATP, of CO_2_ reduction. Finally, the efficiency of the energy transfer, ε_t_, is calculated as

(16) $$\varepsilon_{\text{t}}=\frac{\left(1-y)\;\Delta E_{r}({\text{CO}}_{2}\text{ red}\right)}{-y\;\Delta E_{r}({\text{O}}_{2}\text{ red})},$$

where, as above, *y* and 1–*y* describe the fractions of the total H_2_S pool used for energy generation and CO_2_ reduction, respectively. Note that the values of Δ*E_r_* and Δ*G_r_* in Equations (14–16) are expressed per mole of H_2_S.

As follows from Equations (8, 14–16), the overall thermodynamic efficiency ε_I_ can be expressed as a product of the partial efficiencies of the three steps involved in the energy flow associated with autotrophic sulfur oxidation, namely

(17) $$\varepsilon_{\text{I}}=\varepsilon_{\text{SO}}\;\varepsilon_{\text{u}}\;\varepsilon_{\text{t}}.$$

Because ε_I_ can be calculated from thermodynamic data based on the stoichiometry of autotrophic sulfur oxidation, this means that Equation (17) can be used to calculate any one of the efficiencies ε_SO_, ε_u_ or ε_t_ provided that the other two are known. Although this is not possible at the current state of knowledge, Equation (17) makes it possible to estimate their minimal values. Specifically, the minimal value of ε_SO_ is reached when both ε_u_ and ε_t_ are maximal (i.e., ε_u_ = ε_t_ = 1), which gives ε_SO, I, min_ = ε_I_. Analogously, ε_u, min_ = ε_t, min_ = ε_I_. Thus, ε_I_ represents the minimal value of the partial efficiencies ε_SO_, ε_u_ and ε_t_.

### Efficiency of energy conservation: the new calculation approach

The traditional approach of efficiency calculation does not consider that sulfide oxidation and CO_2_ reduction in SOB are coupled via redox couples such as NAD^+^/NADH, FAD/FADH_2_ and oxidized/reduced Ferredoxin (Fd_ox_/Fd_red_). In our new approach we account for this coupling explicitly. Also, we distinguish between two possible CO_2_ fixation pathways observed in SOB: the Calvin cycle and the reverse tricarboxylic acid (rTCA) cycle. In accordance with Bar-Even et al. (2010), we chose glyceraldehyde-3-phosphate (GA3P) as the primary product of both CO_2_ fixation pathways, even though phosphoenolpyruvate is considered the output of the rTCA cycle (Buchanan and Arnon, 1990). The choice of phosphoenolpyruvate would result in small numerical differences that would, however, not alter significantly our conclusions.

In the SOB employing the Calvin cycle, CO_2_ fixation occurs according to reaction

(18) $$6\text{ NADH}+3\;{\text{CO}}_{2}+{\text{P}}_{\text{i}}\to 6\;{\text{NAD}}^{+}+\text{GA3P}+7\;{\text{H}}_{2}\text{O}.$$

This reaction requires conversion of ATP into ADP and P_i_, and its energy requirement is Δ*G_r_*(CO_2_ fix) = 69.7 kJ CO^−1^_2_ (Supplemental material of Bar-Even et al., 2010). The reducing equivalents NADH required for the reduction of CO_2_ in Equation (18) are produced via membrane-associated reverse electron transport (RET) reactions with zero-valent sulfur (S^0^) or sulfate (SO^2−^_4_) as two possible end products:

(19) $$\;{\text{H}}_{2}\text{S}+{\text{NAD}}^{+}\to S^{0}+\text{NADH}+{\text{H}}^{+},$$

(20) $${\text{H}}_{2}\text{S}+4\;{\text{NAD}}^{+}+4\;{\text{H}}_{2}\text{O}\to {\text{SO}}_{4}^{2-}+4\text{ NADH}+6\;{\text{H}}^{+}.$$

These two endergonic reactions are driven by proton motive force, which is generated either directly by the exergonic reaction given by Equation (3) or by utilizing ATP. When both of them occur simultaneously, the generalized equation for the RET reaction is

(21) $$\begin{array}{l} {\text{H}}_{2}\text{S}+(4-3x)\;{\text{NAD}}^{+}+(4-4x)\;{\text{H}}_{2}\text{O}\to \\ x\;{\text{S}}^{0}+(1-x)\;{\text{SO}}_{4}^{2-}+(4-3x)\text{ NADH}+(6-5x)\;{\text{H}}^{+}, \end{array}$$

where *x* again describes the S^0^: SO^2−^_4_ product ratio, and its energy requirement is calculated as

(22) $$\begin{array}{l} \Delta G_{r}(\text{RET})=x\Delta G_{f}^{0}({\text{S}}^{0})+(1-x)\Delta G_{f}^{0}({\text{SO}}_{4}^{2-})-\Delta G_{f}^{0}({\text{H}}_{2}\text{S})\\ \;-4(1-x)\Delta G_{f}^{0}({\text{H}}_{2}\text{O})+(4-3x)\\ \;[\Delta G_{f}^{0}(\text{NADH})-\Delta G_{f}^{0}({\text{NAD}}^{+})]+\text{RT ln }Q_{3}. \end{array}$$

Here the difference Δ*G*^0^_*f*_(NADH)–Δ*G*^0^_*f*_(NAD^+^) = 60.99 kJ (mol NADH)^−1^ (Alberty, 1998) and the reaction quotient *Q_3_* is given by

(23) $$Q_{3}=\frac{{\left[{\text{SO}}_{4}^{2-}\right]}^{\left(1-x\right)}\;{\left[\text{NADH}\right]}^{\left(4-3x\right)}\;{\left[{\text{H}}^{+}\right]}^{\left(6-5x\right)}}{[{\text{H}}_{2}\text{S}]\;{\left[{\text{NAD}}^{+}\right]}^{\left(4-3x\right)}}.$$

In the SOB employing the rTCA cycle, CO_2_ fixation occurs according to reaction

(24) $$\begin{array}{l} 3\text{ NADH}+4{\text{Fd}}_{\text{red}}+{\text{FADH}}_{2}+3\;{\text{CO}}_{2}\to \\ 3\;{\text{NAD}}^{+}+4\;{\text{Fd}}_{\text{ox}}+\text{FAD}+\text{GA3P}+7{\text{H}}_{2}\text{O}. \end{array}$$

Energy requirement of this reaction is Δ*G_r_*(CO_2_ fix) = 64.3 kJ CO^−1^_2_ (Bar-Even et al., 2010). The reducing equivalents NADH, FADH_2_ and Fd_red_ required for the reduction of CO_2_ in Equation (24) are produced by the reduction of the corresponding redox couples with H_2_S according to two possible reactions:

(25) $$\begin{array}{l} {\text{H}}_{2}\text{S}+0.5\;{\text{NAD}}^{+}+0.67\;{\text{Fd}}_{\text{ox}}+0.17\text{ FAD}\to \\ {\text{S}}^{0}+0.5\text{ NADH}+0.67\;{\text{Fd}}_{\text{red}}+0.17\;{\text{FADH}}_{2}+1.17\;{\text{H}}^{+}, \end{array}$$

(26) $$\begin{array}{l} {\text{H}}_{2}\text{S}+2\;{\text{NAD}}^{+}+2.67\;{\text{Fd}}_{\text{ox}}+0.67\text{ FAD}+4\;{\text{H}}_{2}\text{O}\to \\ {\text{SO}}_{4}^{2-}+2\text{ NADH}+2.67\;{\text{Fd}}_{\text{red}}+0.67\;{\text{FADH}}_{2}+6.67\;{\text{H}}^{+}. \end{array}$$

When both of these reactions occur simultaneously, the generalized equation for the RET reaction is

(27) $$\begin{array}{l} {\text{H}}_{2}\text{S}+(2-1.5x)\;{\text{NAD}}^{+}+(2.67-2x)\;{\text{Fd}}_{\text{ox}}+(0.67-0.5x)\\ \text{ FAD}+(4-4x)\;{\text{H}}_{2}\text{O}\to \\ x\;{\text{S}}^{0}+(1-x)\;{\text{SO}}_{4}^{2-}+(2-1.5x)\text{ NADH}+(2.67-2x)\;{\text{Fd}}_{\text{red}}\\ \;+(0.67-0.5x)\;{\text{FADH}}_{2}+(6.67-5.5x)\;{\text{H}}^{+} \end{array}$$

and its energy requirement is calculated as

(28) $$\begin{array}{l} \Delta G_{r}(\text{RET})=x\Delta G_{f}^{0}({\text{S}}^{0})+(1-x)\Delta G_{f}^{0}({\text{SO}}_{4}^{2-})-\Delta G_{f}^{0}({\text{H}}_{2}\text{S})\\ \;-4(1-x)\Delta G_{f}^{0}({\text{H}}_{2}\text{O})+(2-1.5x)\;\\ \;[\Delta G_{f}^{0}\;(\text{NADH})-\Delta G_{f}^{0}\;({\text{NAD}}^{+})]+(2.67\text{​}-\text{​}2x)\;\\ \;[\Delta G_{f}^{0}\;({\text{Fd}}_{\text{red}})-\Delta G_{f}^{0}\;({\text{Fd}}_{\text{ox}})]+(0.67-0.5x)\\ \;[\Delta G_{f}^{0}\;({\text{FADH}}_{2})\text{​}-\text{​}\Delta G_{f}^{0}\;(\text{FAD})]\text{​}+\text{​RT ln }Q_{4}. \end{array}$$

Here, Δ*G*^0^_*f*_(NADH) − Δ*G*^0^_*f*_(NAD^+^) = 60.99 kJ (mol NADH)^−1^, Δ*G*^0^_*f*_(FADH_2_) − Δ*G*^0^_*f*_(FAD) = 42.65 kJ (mol FADH_2_)^−1^, Δ*G*^0^_*f*_(Fd_red_) − Δ*G*^0^_*f*_(Fd_ox_) = 38.88 kJ (mol Fd_red_)^−1^ (Alberty, 1998) and the reaction quotient *Q_4_* is given by

(29) $$Q_{4}=\frac{\begin{array}{l} {\left[{\text{SO}}_{4}^{2-}\right]}^{\left(1-x\right)}\;{\left[\text{NADH}\right]}^{\left(2-1.5x\right)}\;{\left[{\text{Fd}}_{\text{red}}\right]}^{\left(2.67-2x\right)}\\ {\left[{\text{FADH}}_{2}\right]}^{\left(0.67-0.5x\right)}\;{\left[{\text{H}}^{+}\right]}^{\left(6.67-5.5x\right)} \end{array}}{[{\text{H}}_{2}\text{S}]\;{\left[{\text{NAD}}^{+}\right]}^{\left(2-1.5x\right)}\;{\left[{\text{Fd}}_{\text{ox}}\right]}^{\left(2.67-2x\right)}\;{\left[\text{FAD}\right]}^{\left(0.67-0.5x\right)}}.$$

Taken together, CO_2_ reduction in SOB comprises two steps: production of the reducing equivalents (Equations 21 or 27) and the actual CO_2_ fixation (Equations 18 or 24), as already pointed out by Kelly (1999). Thus, the total energy requirement of CO_2_ reduction is given by the sum of Δ*G_r_*(RET) and Δ*G_r_*(CO_2_ fix). Analogously to Equation (8), in our new approach we therefore calculate the thermodynamic energy conservation efficiency of autotrophic sulfur oxidation as

(30) $$\varepsilon_{\text{II}}=\frac{\left(1-y)\;[\Delta G_{r}(\text{RET})+\Delta G_{r}({\text{CO}}_{2}\text{ fix})\right]}{-y\;\Delta G_{r}({\text{O}}_{2}\text{ red})},$$

where the corresponding Δ*G_r_* values are calculated as described above. It should be noted that ε_II_ > ε_I_ because Δ*G_r_*(CO_2_ red) calculated by the traditional approach (Equation 11) is always lower than the sum Δ*G_r_*(RET) + Δ*G_r_*(CO_2_ fix).

### Factorization of the new energy conservation efficiency

Analogously to the factorization of the traditional thermodynamic efficiency (see Equation 17), the new thermodynamic efficiency ε_II_ defined by Equation (30) can also be written as a product of partial efficiencies characterizing the processes involved in the autotrophic sulfur oxidation. Specifically, because CO_2_ reduction in our new approach is divided into RET and CO_2_ fixation, expression (15) for the efficiency of ATP utilization must be written as

(31) $$\varepsilon_{\text{u},\text{II}}=\frac{\Delta G_{r}(\text{RET})+\Delta G_{r}({\text{CO}}_{2}\text{ fix})}{\Delta E_{r}(\text{RET})+\Delta E_{r}({\text{CO}}_{2}\text{ fix})},$$

where Δ*E_r_*(RET) and Δ*E_r_*(CO_2_ fix) describe the energy requirements, in the form of ATP, of the RET and CO_2_ fixation reactions, respectively. This implies that ε_II_ can be factorized as

(32) $$\varepsilon_{\text{II}}=\varepsilon_{\text{SO}}\;\varepsilon_{\text{u},\text{II}}\;\varepsilon_{\text{t}}.$$

To make the influence of the RET and CO_2_ fixation reactions more explicit, we define their corresponding efficiencies as

(33) $$\varepsilon_{\text{RET}}=\frac{\Delta G_{r}(\text{RET})}{\Delta E_{r}(\text{RET})}$$

and

(34) $$\varepsilon_{\text{CO}2}=\frac{\Delta G_{r}({\text{CO}}_{2}\text{ fix})}{\Delta E_{r}({\text{CO}}_{2}\text{ fix})}.$$

This makes it possible to write ε_II_ as a product

(35) $$\varepsilon_{\text{II}}=\varepsilon_{\text{SO}}\;\varepsilon_{\text{t}}\;\varepsilon_{\text{CO}2}\;\varepsilon_{\text{RET}}\;{\left[\alpha \;\varepsilon_{\text{RET}}+(1-\alpha )\;\varepsilon_{\text{CO}2}\right]}^{-1}$$

where the parameter α is defined as

(36) $$\alpha =\frac{\Delta G_{r}({\text{CO}}_{2}\text{ fix})}{\Delta G_{r}(\text{RET})+\Delta G_{r}({\text{CO}}_{2}\text{ fix})}.$$

Previous work has already characterized the realistic ATP requirements of CO_2_ fixation. Assuming 0% oxygenase activity of RuBisCO, ATP requirements of the Calvin cycle are 3 ATP per CO_2_, whereas ATP requirements of the rTCA cycle are ~1.67 ATP per CO_2_ (Bar-Even et al., 2010). As pointed out by Kelly (1999), these requirements can be considered constant. Assuming the ATP energy content of 41 kJ (mol ATP)^−1^, this translates into Δ*E_r_*(CO_2_ fix) of 123 kJ (mol CO_2_)^−1^ and 68.3 kJ (mol CO_2_)^−1^ for the Calvin and rTCA cycle, respectively. Considering the Δ*G_r_*(CO_2_ fix) values calculated by Bar-Even et al. (2010) (see Section Efficiency of Energy Conservation: The New Calculation Approach), the energy conservation efficiency of the CO_2_ fixation can therefore be considered as a known parameter: ε_CO2_ = 0.57 for the Calvin cycle and ε_CO2_ = 0.94 for the rTCA cycle (Bar-Even et al., 2010). Similarly, the parameter α has also a known value depending on the parameter *x* and on the CO_2_ fixation pathway employed by the SOB, as follows from Equations (22), (28) and (36).

Taken together, this means that although the efficiencies ε_SO_, ε_t_ and ε_RET_ are generally unknown, they are related through Equation (35). This makes it possible to calculate any one of these efficiencies provided that the other two are known. Since this is not possible at the current state of knowledge, Equation (35) can be used to estimate their minimal values. Of specific interest in this study is the minimal value of the efficiency of the sulfur oxidation pathway, ε_SO_. This value is reached when the efficiencies ε_RET_ and ε_t_ are maximal (i.e., ε_RET_ = ε_t_ = 1), which gives

(37) $$\begin{array}{l} \varepsilon_{\text{SO},\text{II},\text{min}}=\varepsilon_{\text{II}}\left[\frac{\alpha }{\varepsilon_{\text{CO}2}}+(1-\alpha )\right]\\ \;=\frac{\left(1-y)\;[\Delta G_{r}(\text{RET})+\Delta E_{r}({\text{CO}}_{2}\text{ fix})\right]}{-y\;\Delta G_{r}({\text{O}}_{2}\text{ red})}, \end{array}$$

as follows from Equations (30), (35) and (36). It should be noted that ε_SO, II, min_ > ε_SO, I, min_. Our new approach therefore provides a more constrained range of possible efficiencies of sulfur oxidation coupled to O_2_ reduction in comparison to the traditional approach.

### Generalized equations and efficiencies for other reduced sulfur oxidation processes

Many SOB can use alternative reduced sulfur species for the reduction of O_2_ and CO_2_, such as thiosulfate. Additionally, in anoxic environments or habitats that are characterized by fluctuating conditions, such as hydrothermal vents, also the reduction of NO^−^_3_ has to be considered as a possible sink of electrons during reduced sulfur oxidation. NO^−^_3_ can either be reduced partially to N_2_ by denitrification or completely to NH^+^_4_ by dissimilatory nitrate reduction to ammonia (DNRA) to generate energy for CO_2_ fixation. The generalized mass-balanced equations for these processes are derived analogously as Equations (1–7) and are given in the Table S1. Furthermore, the corresponding energy conservation efficiencies ε_I_, ε_II_ and ε_SO, II, min_ are calculated from expressions similar to Equations (8), (30), and (37) with the exception that the Δ*G_r_* values in the nominator and denominator are calculated based on the stoichiometry of the corresponding energy-conserving and energy-generating reaction. We implemented these calculations in R (www.cran.r-project.org) as functions and scripts that can be freely downloaded from the internet (http://nanosims.geo.uu.nl/SOX; Supplement 1).

## Results

### Stoichiometry of autotrophic sulfur oxidation from rate measurements

Here we demonstrate how the generic theoretical framework can be used to rapidly evaluate the stoichiometry of autotrophic reduced sulfur oxidation from rate measurements of the reactants involved. Additional examples are provided in Supplement 2.

The first example involves marine *Beggiatoa* strain MS-81-6. Nelson et al. (1986) reported that this strain performed aerobic sulfide oxidation coupled to CO_2_ fixation with the O_2_:ΣH_2_S consumption ratio of ~1.65 and the CO_2_:ΣH_2_S consumption ratio of 0.35. Using Table S1A, these experimental values lead to equations ν_O2_/ν_H2S_ = *y*(2−1.5*x*) = 1.65 and ν_CO2_/ν_H2S_ = (1−*y*)(2−1.5*x*) = 0.35, which yield *y* = 0.825 and *x* = 0. As follows from Table S1A, this implies that (i) the aerobic oxidation of sulfide in this strain was performed according to equation

$$\begin{array}{l} {\text{H}}_{2}\text{S}+1.65\;{\text{O}}_{2}+0.35\;{\text{CO}}_{2}+0.35\;{\text{H}}_{2}\text{O}\to \\ {\text{SO}}_{4}^{2-}+0.35\;{\text{CH}}_{2}\text{O}+2{\text{H}}^{+}, \end{array}$$

(ii) sulfate was the exclusive product, and (iii) 82.5% of the sulfide pool was used in the energy gaining reaction with oxygen (Equation 3) while the remaining 17.5% was used for CO_2_ fixation (Equation 6). This is consistent with the conclusions of Nelson et al. (1986).

The second example deals with SOB that live in symbiosis with tubeworms *Riftia*. Girguis et al. (2002) reported that the sulfide consumption rate of 6.75 μmol ΣH_2_S g^−1^ h^−1^ by the whole-worm symbiosis was accompanied by the total rates of O_2_ and CO_2_ consumption of 12.4 μmol O_2_ g^−1^ h^−1^ and 12.45 μmol CO_2_ g^−1^ h^−1^, respectively. Assuming that host respiration constitutes 25% of the whole-worm O_2_ consumption (see Supplement 3), the estimated rates of O_2_ and CO_2_ consumption by the symbionts are 9.3 μmol O_2_ g^−1^ h^−1^ and 15.55 μmol CO_2_ g^−1^ h^−1^, respectively. This directly translates into equations ν_O2_/ν_H2S_ = *y*(2−1.5*x*) = 9.3/6.75 = 1.38 and ν_CO2_/ν_H2S_ = (1−*y*)(2−1.5*x*) = 15.55/6.75 = 2.3, which yield *x* = −1.12, y = 0.375 and the corresponding equation

$$\begin{array}{l} {\text{H}}_{2}\text{S}+1.12\;{\text{S}}^{0}+1.38\;{\text{O}}_{2}+2.3\;{\text{CO}}_{2}+3.42\;{\text{H}}_{2}\text{O}\to \\ \;2.12\;{\text{SO}}_{4}^{2-}+2.3\;{\text{CH}}_{2}\text{O}+4.24\;{\text{H}}^{+}. \end{array}$$

This means that during the particular experiment reported by Girguis et al. (2002) the *Riftia* symbionts probably oxidized not only H_2_S but also a relatively large amount of stored zero-valent sulfur to fix CO_2_, with 37.5% of this total pool of reduced sulfur used for energy generation. This result is reasonable considering that the sum of the O_2_ and CO_2_ consumption rates cannot be larger than double the sulfide consumption rate if aerobic sulfide oxidation is performed with H_2_S and SO^2−^_4_ as the only reduced and oxidized sulfur species, respectively (see Table S1A).

### Energy conservation efficiency from stoichiometry

The energy conservation efficiency of autotrophic sulfur oxidation can be calculated once the stoichiometry of the generalized equation is known. First, we demonstrate this calculation for *Beggiatoa* strain MS-81-6 analyzed above using the traditional calculation approach and assuming standard biochemical conditions. Substitution of *x* = 0 and of the Δ*G*^0^_*f*_ values tabulated in Thauer et al. (1977) to Equations (10) and (11) yields Δ*G^0^_r_*(O_2_ red) = −829 kJ mol^−1^ and Δ*G^0^_r_*(CO_2_ red) = 145 kJ mol^−1^. Subsequent substitution of these values together with *y* = 0.825 obtained above to Equation (8) yields ε_I_ = 0.037. This is consistent with the efficiency value of 0.038 obtained by Nelson and Hagen (1995). The small discrepancy is most likely due to the fact that Nelson and Hagen (1995) used Δ*G*^0^_*f*_(O_2_) = 0 kJ mol^−1^ for the standard Gibbs energy of formation of O_2_, which corresponds to O_2_ gas, while we used the value of 16.4 kJ mol^−1^, which corresponds to dissolved O_2_.

Traditionally, the efficiency value of 0.037 is interpreted such that *Beggiatoa* strain MS-81-6 conserves 3.7% of the energy gained by aerobic sulfide oxidation into biomass. Based on our analysis above (Equation 17), a more accurate interpretation is that 0.037 is the *product* of the partial efficiencies of (i) ATP generation by O_2_ reduction (ε_SO_), (ii) ATP utilization for CO_2_ fixation (ε_u_) and (iii) the transfer of ATP gained by O_2_ reduction for CO_2_ fixation and thus not for cellular maintenance (ε_t_). For example, an assumption that *Beggiatoa* strain MS-81-6 uses ATP exclusively for CO_2_ fixation and not for any other biochemical reaction (ε_t_ = 1), and that its ATP utilization for CO_2_ fixation occurs without loss of energy (ε_u_ = 1), would imply that the efficiency of ATP generation by O_2_ reduction (see Equation 14) in this strain is ε_SO_ = ε_I_/(ε_t_ε_u_) = 0.037.

In the second example we analyse the difference between the efficiency values calculated according to the traditional (ε_I_; Equation 8) and our new approach (ε_II_; Equation 30). We do this for aerobic thiosulfate oxidizers *Sulfurimonas denitrificans* and *Thioalkalivibrio versutus*, which fix CO_2_ using the rTCA cycle (Hügler et al., 2005) and the Calvin cycle, respectively. Based on the substrate consumption rates reported by Hoor (1981) and Sorokin et al. (2001) we calculated *x* = 0 and *y* = 0.833 for *S. denitrificans* and *x* = 0 and *y* = 0.844 for *T. versutus* (see Supplement 4). Using these values in the traditional approach, we obtained the efficiency of ε_I_ = 0.0314 for *S. denitrificans* and ε_I_ = 0.0289 for *T. versutus*, whereas our new approach gave values ε_II_ = 0.0799 for *S. denitrificans* and ε_II_ = 0.0796 for *T. versutus*.

We see that our new approach yields substantially larger efficiency values than the traditional approach. This is generally because the new approach accounts for the fact that CO_2_ reduction by SOB is performed in two separate steps (i.e., the reduction of an electron carrier via reverse electron transport followed by the reduction of CO_2_ coupled to the electron carrier oxidation in the respective CO_2_ fixation pathway) and because the theoretical energy requirements of these two steps [Δ*G_r_*(RET) + Δ*G_r_*(CO_2_ fix); see Equation (30)] are substantially larger than the theoretical energy requirement of the net CO_2_ reduction reaction [Δ*G_r_*(CO_2_ red); see Equation (8)] considered in the traditional approach. Additionally we see that the relative difference between the efficiency values of the compared SOB depends on the choice of the approach, particularly if the SOB employ different CO_2_ fixation pathways. In this specific case the efficiency ε_I_ of *S. denitrificans* is by about 8% larger than that of *T. versutus*, whereas their ε_II_ values are practically identical. This is related to the fact that the Δ*G_r_* values of the RET and CO_2_ fixation reactions (Equation 30) depend on the type of the electron carriers involved in the CO_2_ fixation pathways, which differ between the rTCA and Calvin cycle (compare Equations 18 and 21 vs. Equations 24 and 27).

As a last point, we illustrate an additional insight that can be gained from the factorized form of ε_II_ (see Equation 35). The fact that *S. denitrificans* and *T. versutus* have practically identical values of the overall efficiency ε_II_ might be misinterpreted by concluding that their corresponding partial efficiencies ε_SO_, ε_RET_, ε_CO2_, and ε_t_ are also the same. However, we know that the efficiency ε_CO2_ of the rTCA cycle used by *S. denitrificans* to fix CO_2_ is almost two-fold larger than that of the Calvin cycle employed by *T. versutus*. This implies that at least one of the efficiencies ε_SO_, ε_RET_, and ε_t_ must differ between the two SOB. By calculating the minimum values of these efficiencies using Equations (35–37) we found that all of them are higher for *T. versutus* (ε_SO, II, min_ = ε_t, II, min_ = 0.1030 and ε_RET, II, min_ = 0.052) than for *S. denitrificans* (ε_SO, II, min_ = ε_t, II, min_ = 0.0818 and ε_RET, II, min_ = 0.051). This suggests that *T. versutus* has a more efficient sulfur oxidation pathway (larger ε_SO_ or ε_RET_) or lower cellular maintenance requirements (larger ε_t_) than *S. denitrificans*. It should be noted that this conclusion can only be drawn based on our new calculation approach but not based on the traditional approach. Therefore, our new approach, and particularly the value of ε_SO, II, min_, is better suited to identify differences between sulfur oxidation pathways among SOB, particularly if the compared SOB employ different CO_2_ fixation pathways.

### Sensitivity of the calculated efficiency toward reactant concentrations

Until now our calculations assumed standard biochemical conditions. However, as pointed out by Nelson and Hagen (1995) and Kelly (1999), the actual experimental conditions should be used when calculating the Δ*G_r_* values for the energy gaining and energy conserving reactions. In the literature on SOB activity these concentrations are in most cases not reported or not well constrained. Thus, it is important to evaluate possible errors introduced by this uncertainty in the calculation of the energy conservation efficiency. This is achieved through sensitivity analysis.

To perform this analysis, we varied the concentration of one of the reactants between 1 M and 1 nM, or the pH between 2 and 12, while keeping the other reactant concentrations as well as the stoichiometry of the reaction mediated by an SOB unchanged. In our formalism the variations of the reactant concentrations and pH are captured in the variation of the quotients *Q* (see Equations 12, 13, 23, and 29). As examples, we used the aerobic sulfide oxidizing bacteria *Thermithiobacillus tepidarius* and *Halothiobacillus neapolitanus*, which employ the Calvin cycle for CO_2_ fixation, and the denitrifying thiosulfate oxidizing bacteria *Sulfurimonas hongkongensis* and *Sulfurimonas denitrificans*, which employ the rTCA cycle for CO_2_ fixation.

This analysis revealed that the calculated efficiency values are most sensitive to the concentrations of CO_2_ (ε_I_) and of the electron carriers such as NAD^+^/NADH (ε_SO, II, min_), whereas the sensitivity toward the concentration of the terminal electron acceptor (TEA) is lowest (Figure S2). Sensitivities toward pH, the reduced sulfur compound (e.g., S_2_O_3_^2−^ or H_2_S) and SO^2−^_4_ (Figure S2) as well as toward temperature between 0 and 30°C (data not shown) are intermediate but also rather small. For instance, for the aerobic sulfide oxidizing bacteria *T. tepidarius* and *H. neapolitanus* a change in the concentration of NAD^+^ by three orders of magnitude would change the calculated value of ε_SO, II, min_ by ~14%, while the same change in the concentration of CO_2_ would change ε_I_ by ~24%. In contrast the calculated values of ε_SO, II, min_ and ε_I_ would only change by ~4% if the concentration of O_2_ changed by three orders of magnitude. The errors introduced by uncertainties in the reactant concentrations are similar for the denitrifying thiosulfate oxidizing bacteria *S. hongkongensis* and *S. denitrificans* (Figure S2).

As the aim of this study is to compare efficiencies among SOB, it is important to evaluate how the errors introduced by the uncertainties in the reactant concentrations would affect this comparison. To do this we considered two SOB whose efficiencies calculated at standard conditions differ, and determined the change in the reactant concentrations required to make the efficiencies equal. We illustrate the result on the same pairs of aerobic and anaerobic SOB as above. When using the traditional approach, the efficiency ε_I_ calculated for *T. tepidarius* would become equal to that of *H. neapolitanus* if the former were calculated with a 10^6^-fold lower SO^2−^_4_ concentration than the standard concentration of 1 M or at pH = 10 instead of pH = 7. In contrast, the difference in the SO^2−^_4_ concentration or pH would need to be considerably larger (~10^20^-fold for SO^2−^_4_, or at a pH≈11) to achieve the same effect for the efficiency ε_SO, II, min_ calculated by our new approach (Figure S2A). Similar conclusion can be drawn for *S. hongkongensis* and *S. denitrificans* (Figure S2B) as well as for SOB that perform other types of autotrophic sulfur oxidation (data not shown). We therefore conclude that, although the sensitivities toward reactant concentrations are similar for both approaches, our new approach is more robust to resolve differences between SOB with respect to their energy conservation efficiency if the calculations are affected by an uncertainty in the concentrations of the reactants involved.

### Efficiency of autotrophic sulfur oxidation in different strains of SOB

The reason for different efficiencies of autotrophic sulfur oxidation is generally assumed to lie in the different biochemical pathways that the SOB employ for the oxidation of the reduced sulfur compound (e.g., Hagen and Nelson, 1997; Kelly, 1999). To gain more insights into this possible relationship, we compiled literature data on cultivated strains of SOB, calculated their energy conservation efficiencies, and listed them together with the available information on the identified sulfur oxidation and carbon fixation pathways and selected specific enzymes. To follow the outcomes of the above analyses, we used ε_SO, II, min_ to compare the SOB. Specifically, we first calculated the complete stoichiometry of sulfur oxidation coupled to TEA and CO_2_ reduction based on rates reported in the literature. As for most SOB these rates were obtained under not well-constrained experimental conditions, we could not include the concentration dependency of the Δ*G_r_* values (in the form of the reaction quotient *Q*) in our efficiency calculations. Thus, to still make a comparison possible, we chose to calculate the efficiency values at standard biochemical conditions.

**Figure 2:**
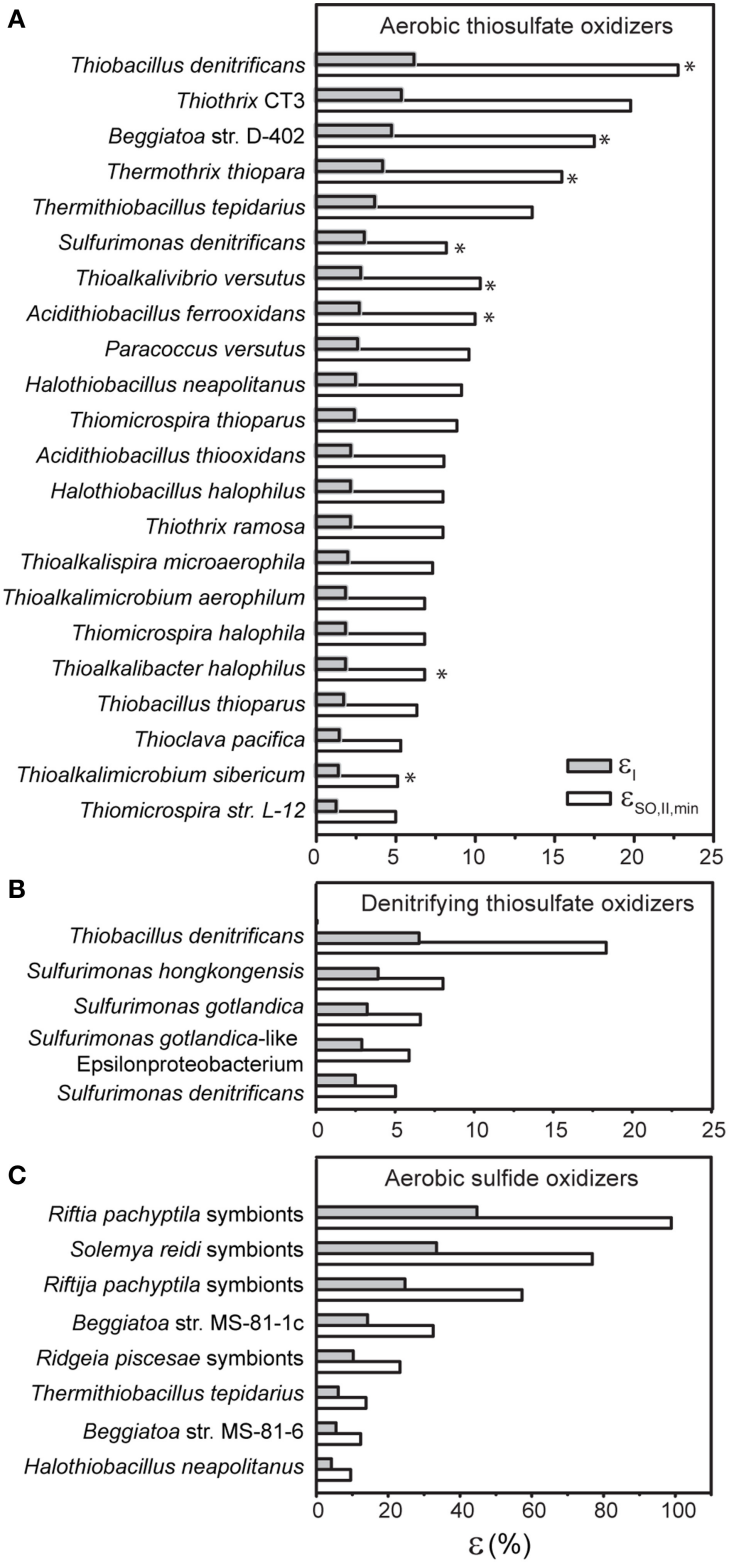
**Energy conservation efficiencies of SOB performing aerobic thiosulfate oxidation (A), thiosulfate oxidation coupled to denitrification (B) and aerobic sulfide oxidation (C)**. Values were calculated using experimental data in the literature (see Table 1) based on the traditional approach (ε_I_) and our new approach (ε_SO, II, min_). Asterisks in panel A indicate that the SOB are facultatively anaerobic.

The results of this compilation are summarized in Table 1 and Figure 2. First, they show that the efficiencies cover a wide range with no apparent clustering according to the SOB phylogeny. Furthermore, they suggest possible links between the efficiency, the sulfur oxidation pathway and/or the type of TEA reductase. This is quite astonishing considering that the efficiency values were calculated from rate measurements performed under different experimental settings (e.g., batch reactor studies, continuous cultivation) and that coherent data on both stoichiometry and biochemical pathways employed by SOB are rather limited.

**Table 1:** **Characteristics of autotrophic sulfur oxidizing bacteria (SOB) derived from literature data and calculated in this study (ε_I_, ε_II_, ε_SO, II, min_, CO_2_:TEA)**.

		***x***	***y***	**ε_I_ (%)^a^**	**ε_II_ (%)^b^**	**ε_SO, II, min_(%)^c^**	**CO_2_:TEA^d^**	**PB^e^**	**C-fixation pathway**		**Sulfur oxidation pathways**				**Selected enzymes**				**Ref^l^**
									**Calvin^f^**	**rTCA^g^**	**Branched**	**Sox**	**SI_4_**	**APS**	**rDSR^h^**	**SDO^i^**	**Nar^j^**	**Nap^k^**	
Aerobic thiosulfate oxidizers	*Thiobacillus denitrificans*	0	0.710	6.39	17.59	22.76	0.41	β	X^m^		X		X	X	X				1–6
		0	0.740	5.50	15.13	19.58	0.35												
	*Thiothrix* CT3	0	0.738	5.55	15.29	19.78	0.36	γ	X		X								7
	*Beggiatoa* str. D-402	0	0.761	4.91	13.52	17.50	0.31	γ	X		X		X		X				8
	*Thermothrix thiopara*	0	0.783	4.34	11.93	15.44	0.28	β	X										9, 10
	*Thermithiobacillus tepidarius*	0	0.804	3.81	10.50	13.58	0.24	γ	X				X						5, 11, 12
	*Thioalkalivibrio versutus*	0	0.844	2.90	7.96	10.30	0.18	γ	X				X						13, 14
	*Acidithiobacillus ferrooxidans*	0	0.848	2.80	7.72	9.99	0.18	γ	X				X			X			3, 16, 17, 18
	*Paracoccus versutus*	0	0.853	2.70	7.42	9.60	0.17	α	X			X							10, 15
	*Halothiobacillus neapolitanus*	0	0.859	2.57	7.07	9.15	0.16	γ	X				X	X		X			3, 19, 20
	*Thiomicrospira thioparus*	0	0.863	2.48	6.84	8.85	0.16	β	X				X	X					20, 21
	*Sulfurimonas denitrificans*	0	0.833	3.13	7.99	8.18	0.20	ε		X		X							6, 39, 40
	*Acidithiobacillus thiooxidans*	0	0.874	2.26	6.21	8.03	0.14	γ	X				X	X		X			10, 22–24
	*Halothiobacillus halophilus*	0	0.875	2.23	6.15	7.96	0.14	γ	X				X						25–27
	*Thiothrix ramosa*	0	0.875	2.23	6.15	7.96	0.14	γ	X		X			X					5, 28
	*Thioalkalispira microaerophila*	0	0.884	2.05	5.65	7.31	0.13	γ	X										14, 29
	*Thioalkalimicrobium aerophilum*	0	0.891	1.91	5.27	6.82	0.12	γ	X				X						13
	*Thiomicrospira halophila*	0	0.891	1.91	5.27	6.82	0.12	γ	X			X?	X						5, 30
	*Thioalkalibacter halophilus*	0	0.891	1.91	5.27	6.82	0.12	γ	X			X?							5, 31
	*Thiobacillus thioparus*	0	0.898	1.78	4.89	6.33	0.11	β	X		X		X	X	X	X			5, 17, 32–36
	*Thioclava pacifica*	0	0.913	1.49	4.10	5.31	0.10	α	X			X?							37
	*Thioalkalimicrobium sibericum*	0	0.916	1.43	3.95	5.11	0.09	γ	X			X?							13
	*Thiomicrospira sp. Strain L-12*	0.2	0.921	1.29	3.91	4.99	0.09	γ	X			X	X						38, 39
Denitrifying thiosulfate oxidizers	*Thiobacillus denitrificans*	0	0.775	5.15	14.18	18.35	0.36	β	X		X		X	X	X		X		1, 4, 6
		0	0.812	4.11	11.31	14.63	0.29												40
	*Sulfurimonas hongkongensis*	0	0.852	3.08	7.85	8.04	0.22	ε		X		X							41
	*Sulfurimonas gotlandica*	0	0.875	2.53	6.45	6.61	0.18	ε		X		X		X			X	X	42, 43
	*Sulfurimonas gotlandica-*like Epsilonproteobacterium (environmental sample)	0	0.887	2.26	5.76	5.89	0.16	ε		X		X							43, 44
	*Sulfurimonas denitrificans*	0	0.902	1.93	4.91	5.03	0.14	ε		X		X						X	6, 45
Aerobic sulfide oxidizers	*Riftia pachyptila* symbionts^n^	−1.12	0.375	30.52	77.27	98.86	1.67	γ	X	X	X			X	X				46, 47, 48
	*Solemya reidi* symbionts	0.17	0.429	22.83	59.80	76.87	1.33	γ	X		X	X		X	X				49, 50
	*Riftia pachyptila* symbionts^n^	0.33	0.500	16.80	44.50	57.29	1	γ	X	X	X			X	X				5, 46, 48, 51
	*Beggiatoa* str. MS-81-1c	0.05	0.641	9.72	25.30	32.50	0.56	γ	X					X					52
	*Ridgeia piscesae* symbionts	0	0.714	6.98	18.13	23.29	0.40	γ	X					X					53
	*Thermithiobacillus tepidarius*	0	0.808	4.14	10.76	13.81	0.24	γ	X				X						12, 54
	*Beggiatoa* str. MS-81-6^n^	0	0.825	3.70	9.60	12.33	0.21	γ	X				X						52
	*Halothiobacillus neapolitanus*	0	0.859	2.86	7.43	9.54	0.16	γ	X			X	X			X			20, 53, 51

a ε_I_ is the efficiency of energy conservation calculated at biochemical standard conditions, i.e., 1 M concentration of all reactants, 25°C, pH 7, according to the traditional approach.

b ε_II_ is the efficiency of energy conservation calculated at biochemical standard conditions according to our new approach.

c ε_SO, II, min_ is the minimum efficiency of the sulfur oxidation coupled to TEA reduction calculated at biochemical standard conditions based on our new approach.

d TEA is the terminal electron acceptor in the energy generating reaction (O_2_ in aerobic processes or NO^−^_3_ in denitrification). The complete equations for the corresponding autotrophic sulfur oxidation reactions are given in Table S2.

e PB refers to the proteobacterial class.

f Calvin refers to the Calvin-Benson-Bassham cycle.

g rTCA refers to the reverse tricarboxilic acid cycle.

h rDSR refers to the reverse dissimilatory sulfite reductase.

i SO refers to sulfur dioxygenase.

j Nar refers to the membrane-bound nitrate reductase system.

k Nap refers to the periplasmic nitrate reductase system.

l References: 1, (Justin and Kelly, 1978); 2, (Aminuddin, 1980); 3, (Kelly, 1999); 4, (Beller et al., 2006); 5, (Meyer et al., 2007); 6, (Hoor, 1981); 7, (Rossetti, 2003); 8, (Grabovich et al., 2001); 9, (Brannan and Caldwell, 1983); 10, (Mason et al., 1987); 11, (Lu and Kelly, 1988); 12, (Wood and Kelly, 1986); 13, (Sorokin et al., 2001); 14, (Sorokin et al., 2013); 15, (Friedrich et al., 2001); 16, (Eccleston and Kelly, 1978); 17, (Rohwerder and Sand, 2003); 18, (Pronk et al., 1990); 19, (Hempfling and Vishniac, 1967); 20, (Kelly, 1982); 21, (Lengeler et al., 2009); 22, (Masau et al., 2001); 23, (Kamimura et al., 2005); 24, (Nakamura et al., 2014); 25, (Wood and Kelly, 1991); 26, (Kelly and Wood, 2000); 27, (Shi et al., 2011); 28, (Odintsova et al., 1993); 29, (Sorokin, 2002); 30, (Sorokin et al., 2006); 31, (Banciu et al., 2008); 32, (Smith and Kelly, 1988); 33, (Peck, 1960); 34, (Suzuki, 1974); 35, (Trüper, 1982); 36, (Loy et al., 2009); 37, (Sorokin et al., 2005); 38, (Ruby and Jannasch, 1982); 39, (Sievert et al., 2007); 40, (Schedel et al., 1975); 41, (Cai et al., 2014); 42, (Bruckner et al., 2013); 43, (Grote et al., 2012); 44, (Brettar et al., 2006); 45, (Sievert et al., 2008); 46, (Nelson and Hagen, 1995); 47, (Girguis et al., 2002); 48, (Markert et al., 2007); 49, (Anderson et al., 1987); 50, (Stewart et al., 2011); 51, (Girguis and Childress, 2006); 52, (Hagen and Nelson, 1997); 53, (Nyholm et al., 2008); 54, (Kelly et al., 1993); 55, (Veith et al., 2012); 56, (Kelly et al., 1997).

m “X” indicates the presence of a pathway or enzyme; “X?” indicates that the presence of a pathway or enzyme is likely but not conclusively confirmed.

n ε_II_ and ε_SO, II, min_ were calculated based on the assumption that the symbiotic SOB employ the Calvin cycle for CO_2_ fixation.

With regard to aerobic thiosulfate oxidizing bacteria, for which the available literature data is most abundant, our analysis shows that autotrophic thiosulfate oxidation measured under aerobic conditions appears to be performed with the highest efficiency if the SOB are facultatively anaerobic (indicated by asterisk in Figure 2), a trend noticed already, for instance, by Kelly (1982). Concerning the pathways of thiosulfate oxidation, SOB equipped with the enzyme reverse dissimilatory sulfite reductase (rDSR), which is involved in the branched pathway (red arrows in Figure 1), tend to have the highest efficiencies among the aerobic thiosulfate oxidizers, whereas those relying on the Sox pathway (green arrows in Figure 1) appear to have the lowest efficiencies. In contrast, no clear patterns are apparent for the SOB employing the SI_4_ pathway (blue arrows in Figure 1), whose efficiencies span from low to high values. The same is true for the APS pathway for sulfite oxidation, which can be part of both the branched and SI_4_ pathways (Figure 1).

Aerobic sulfide oxidizing bacteria employing pathways that involve oxidation of zero-valent sulfur (black arrows in Figure 1) to sulfite via rDSR (red arrows in Figure 1) have the highest efficiency (Table 1). This is similar to the pattern identified for the aerobic thiosulfate oxidizing bacteria. Most strikingly, extremely high efficiencies of energy conservation are found among aerobic sulfide oxidizers that live in symbiotic association with invertebrates such as the *Riftia* tubeworm. Note that these high values are not the consequence of the fact that they were calculated at standard biochemical conditions. Specifically, the concentrations of the substrates and products involved would have to be in the kilomolar and picomolar range, respectively, to obtain efficiency values comparable to those of the other, less efficient SOB. Since it is unlikely that the host regulates the substrates and products in such an extreme range of concentrations, the high efficiency values of the symbiotic SOB are realistic.

With respect to SOB that couple thiosulfate oxidation to denitrification, the data available in the literature is very limited. Nevertheless, the present data indicate that SOB equipped with a membrane-bound nitrate reductase system (Nar) have in general high efficiencies whereas those relying exclusively on a periplasmic nitrate reductase system (Nap), namely *S. denitrificans*, have a very low energy conservation efficiency (Table 1).

### Stoichiometry from the efficiency of energy conservation and limited rate measurements

Most stoichiometries and efficiency values listed in Table 1 and Table S2 were calculated based on rate measurements during which the studied SOB oxidized the sulfur compound completely to sulfate. However, for many SOB main products of sulfur oxidation may include also other sulfur species, such as zero-valent sulfur (e.g., Ghosh and Dam, 2009). To calculate the stoichiometry of autotrophic sulfur oxidation at an arbitrary S^0^:SO^2−^_4_ production ratio, one generally needs to provide gross rates of three reactants involved. However, accurate quantification of three gross rates is often experimentally difficult. Here we demonstrate that the stoichiometry of autotrophic sulfur oxidation can be estimated if the consumption/production rates of only two reactants involved are measured, provided that certain additional conditions characterizing the activity of an SOB are known.

Our framework shows that the stoichiometry is completely determined by the values of the parameters *x* and *y*. To find these values, two constraints are needed. The first constraint is obtained by measuring the rates of two reactants involved, whereby their ratio directly provides a relationship between *x* and *y*, as follows from the generalized mass balanced equations in Table S1. The second constraint is obtained from the knowledge of how the CO_2_:TEA consumption ratio (essentially the parameters *y*; see Table S1) or the energy conservation efficiency (e.g., ε_II_) varies depending on the S^0^:SO^2−^_4_ production ratio, i.e., on the parameter *x*. Unfortunately, such information is presently not available because, to the best of our knowledge, the complete stoichiometry of autotrophic sulfur oxidation has never been measured over variable *x* in an isolated SOB. Therefore, to illustrate the general approach based on our generic framework, we first assume that either the CO_2_:TEA ratio or the efficiency ε_II_ is independent of *x* and equal to the value calculated from the known stoichiometry at *x* = 0 (see Table 1). We use the aerobic sulfide oxidizing *Beggiatoa* str. MS-81-6 as an example.

Based on the experimental data reported by Nelson et al. (1986) we showed above that this SOB performs aerobic sulfide oxidation to SO^2−^_4_ (i.e., *x* = 0) with the CO_2_:O_2_ consumption ratio of 0.21, O_2_:ΣH_2_S ratio of 1.65 and, when accounting for the energy requirements of the CO_2_ fixation pathway, with the efficiency of ε_II_ = 9.60% (Table 1 and Table S2). Let us now suppose that in a separate experiment the O_2_:ΣH_2_S consumption ratio by this strain would be 0.5. What would be the corresponding stoichiometry?

As follows from Table S1A, the generalized stoichiometric coefficients must be related as ν_O2_/ν_H2S_ = O_2_:ΣH_2_S = 0.5, which yields the first relationship between *x* and *y*, namely *y*(2−1.5*x*) = 0.5. If we additionally assume that the energy conservation efficiency ε_II_ is independent of *x* and equal to the value at *x* = 0 (i.e., 9.60%; Table 1), the second relationship between *x* and *y* is provided by Equation (30). By solving these two equations numerically (implemented in a script written in R available on the internet at http://nanosims.geo.uu.nl/SOX; Supplement 1) we obtained *x* = 0.920 and *y* = 0.807 and thus the stoichiometry

$$\begin{array}{l} {\text{H}}_{2}\text{S}+0.5\;{\text{O}}_{2}+0.12\;{\text{CO}}_{2}\to \\ 0.08\;{\text{SO}}_{4}^{2-}+0.92\;{\text{S}}^{0}+0.12\;{\text{CH}}_{2}\text{O}+0.8\;{\text{H}}_{2}\text{O}+0.16\;{\text{H}}^{+}. \end{array}$$

As mentioned above and applied in the work of Nelson et al. (1986), the alternative approach assumes that the growth yield per mole of TEA utilized is independent of *x* and equal to the value determined at *x* = 0 (i.e., CO_2_:O_2_ = 0.21). Using the generalized stoichiometric coefficients (see Table S1), this implies ν_CO2_/ν_O2_ = (1−*y*)/*y* = 0.21 and thus the value of *y* = 0.826. Combination of this value with the first constraint (i.e., *y*(2–1.5*x*) = 0.5; see above) then yields *x* = 0.929 and thus the stoichiometry

$$\begin{array}{l} {\text{H}}_{2}\text{S}+0.5\;{\text{O}}_{2}+0.11\;{\text{CO}}_{2}\to \\ 0.07\;{\text{SO}}_{4}^{2-}+0.93\;{\text{S}}^{0}+0.11\;{\text{CH}}_{2}\text{O}+0.82\;{\text{H}}_{2}\text{O}+0.14\;{\text{H}}^{+}. \end{array}$$

**Figure 3:**
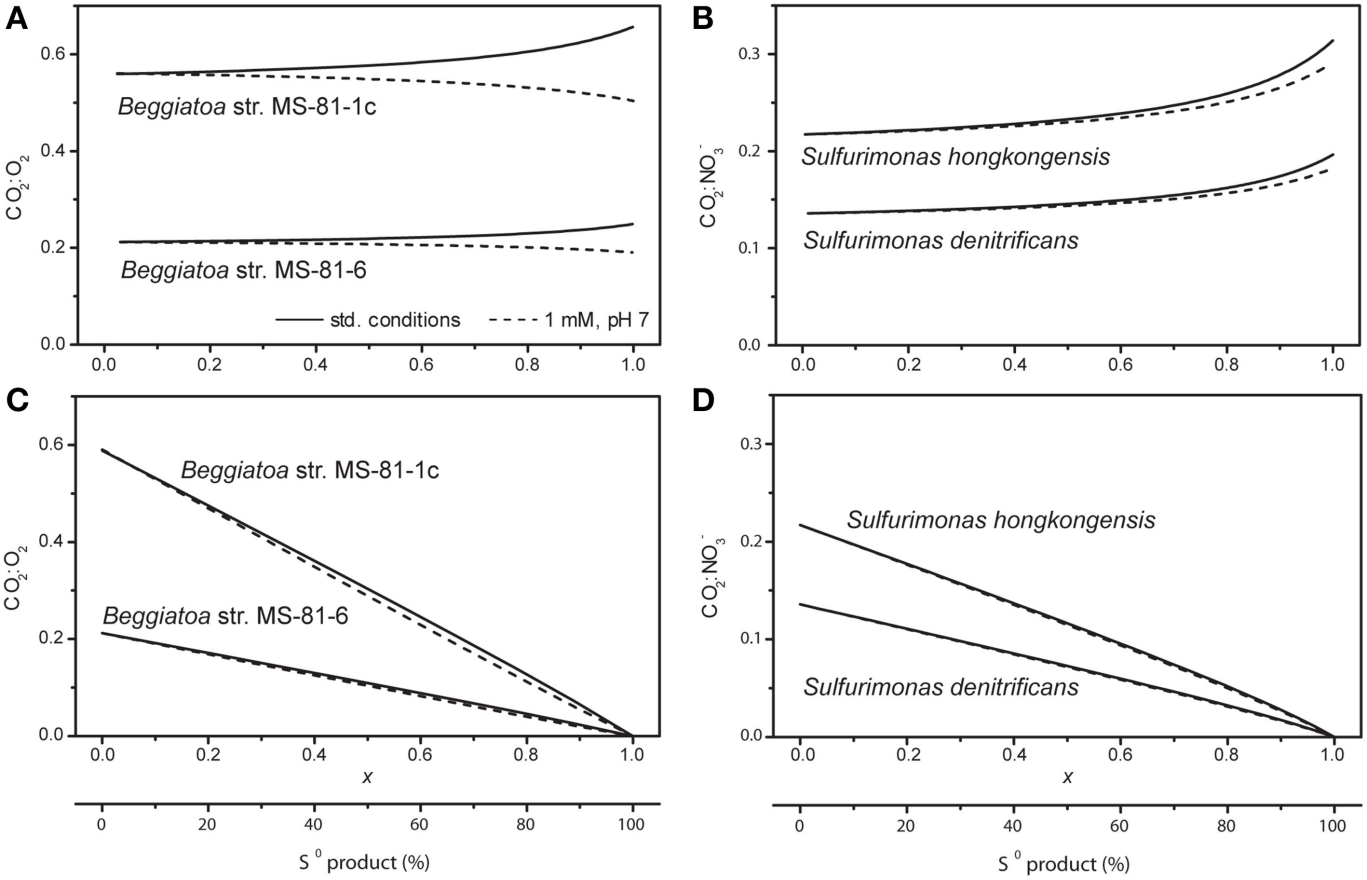
**CO_2_:TEA ratios in selected SOB calculated over the complete range of S^0^:SO^2−^_4_ production ratios (represented by 0<*****x*****<1)**. Calculations were done for two aerobic **(A,C)** and two anaerobic **(B,D)** SOB at standard biochemical conditions (solid lines) and with all reactant concentrations equal to 1 mM and pH = 7 (dashed lines), assuming that the energy conservation efficiency ε_II_ is constant **(A,B)** or linearly decreasing from the maximum reached at *x* = 0 toward zero reached at *x* = 1 **(C,D)**.

We see that the efficiency-based and CO_2_:TEA-based approach give a very similar stoichiometry of the overall sulfide oxidation reaction, suggesting that they are almost equivalent. To verify this, we performed the same calculations over all possible values of *x* as well as for different aerobic and anaerobic SOB. If the two approaches were equivalent, the calculated CO_2_:TEA consumption ratios would not change with *x.* As shown in Figures 3A,B, this is approximately the case, since the calculated CO_2_:TEA ratios vary by less than ~20% over the complete interval of *x.* Additionally, this variability is decreased if the reactant concentrations are decreased from values at standard biochemical conditions to a more environmentally relevant range (compare solid and dashed lines in Figures 3A,B). This means that the possible error made when determining the stoichiometric coefficients (for an arbitrary value of *x*) based on the CO_2_:TEA-based approach or the efficiency-based approach will be relatively small (<20%).

The assumption that either the CO_2_:TEA ratio or the efficiency ε_II_ is independent of *x* is likely not generally applicable to all SOB. Indeed, gslund (Høgslund et al. 2009) showed that sulfide oxidation to S^0^ in bundles of *Thioploca* spp. is completely decoupled from CO_2_ fixation (CO_2_:TEA = 0), implying *y* = 1 and ε_II_ = 0 at *x* = 1. It is therefore likely that in some SOB the variability of the CO_2_:TEA ratio or of the efficiency ε_II_ with the parameter *x* is better approximated by a linear function that, for instance, reaches a maximum at *x* = 0 and some other value, such as zero, at *x* = 1. By assuming that this is the case for the efficiency ε_II_, we found that the calculated CO_2_:TEA ratio also very closely follows a linear trend (Figures 3C,D). This means that, again, the possible error made when determining the stoichiometric coefficients (for an arbitrary value of *x*) based on the CO_2_:TEA-based approach or the efficiency-based approach will be very small.

Overall, these examples demonstrate how our framework can be applied to calculate the stoichiometry of autotrophic sulfur oxidation reactions from two experimentally determined rates (e.g., the consumption rates of the oxidant and of the reduced sulfur species) at an arbitrary S^0^:SO^2−^_4_ production ratio. Prerequisite for such calculations is the knowledge of a function that describes the dependency of the CO_2_:TEA yield or of the energy conservation efficiency on the S^0^:SO^2−^_4_ production ratio parameterized by *x*. When, due to the lack of experimental data, these functions need to be approximated based on experiments performed at only one or two values of *x*, the results obtained from the CO_2_:TEA-based approach and the efficiency-based approach will be very similar.

## Discussion

### Calculation of energy conservation efficiency in SOB

The aim of calculating the energy conservation efficiency is to gain insights into the relationship between the ATP requirements and ATP yields of microbially mediated processes (Baas-Becking and Parks, 1927; Hoor, 1981). For chemolithoautotrophic sulfur oxidation the efficiency is calculated as the ratio between the Gibbs free energy required to fix CO_2_ and the Gibbs free energy gained from the oxidation of the reduced inorganic sulfur compound (Kelly, 1982, 1990). However, this ratio is generally difficult to interpret because of the complexity of the ATP-consuming and ATP-producing pathways involved. Specifically, ATP is gained during the transfer of electrons from the reduced sulfur compound to the TEA (e.g., O_2_ or NO^−^_3_) in the diverse sulfur oxidation pathways (Figure 1) and additionally during substrate-level phosphorylation (e.g., in the APS pathway, Figure 1). In contrast, ATP is required during the reverse electron transport from the sulfur compound to the electron carrier such as NAD^+^ (Elbehti et al., 2000), the reduction of CO_2_ by the electron carrier in the carbon fixation pathway, and for all other biochemical pathways associated with cell maintenance. Since each of the enzymes involved in the diverse steps of sulfur compound oxidation has a specific electron acceptor in the electron transport chain, the amount of proton-motive-force, and thus ATP, generated per electron transferred depends on the level at which the electron enters the transport chain and the type of TEA reductase. Similarly, the ATP requirement of the electron carrier reduction depends on the level at which electrons enter the transport chain. Last but not least, ATP requirements of the CO_2_ fixation also depend on the specific carbon fixation pathway used.

The traditional calculation approach of the energy conservation efficiency is based on the Gibbs free energy of the *net* reactions involved in energy consumption (Equation 6) and energy generation (Equation 3) (Nelson and Hagen, 1995; Kelly, 1999). Since this approach does not consider the above-mentioned complexity of the pathways involved in the autotrophic sulfur oxidation, especially the influence of the CO_2_ fixation pathway and reverse electron transport reactions, it cannot identify which biochemical pathway or reaction has the highest impact on the calculated efficiency. In contrast, our new approach makes this differentiation at least partially possible. Specifically, the calculated value of ε_II_ represents the overall energy conservation efficiency of sulfur oxidation that accounts for the efficiency of the CO_2_ fixation pathway employed by the SOB. Additionally, by formulating this overall efficiency as a product of partial efficiencies of sulfur oxidation coupled to TEA reduction (ε_SO_), energy utilization for reverse electron transfer and CO_2_ fixation (ε_RET_ and ε_CO_2__) and the “transfer efficiency” (ε_t_) (see Equation 35 and Figure S1B), our new approach makes it possible to constrain the range of values that these partial efficiencies can have. Of particular utility is the minimum efficiency of sulfur oxidation coupled to TEA reduction (ε_SO, II, min_), which allows the comparison of SOB with respect to the efficiency of their sulfur oxidation pathways independent of the type and efficiency of their CO_2_ fixation pathway.

### Variability of energy conservation efficiencies amongst SOB

Possible links between the energy conservation efficiency and pathways of sulfur oxidation have been extensively discussed before (Kelly, 1982, 1999, 2003; Nelson and Hagen, 1995; Hagen and Nelson, 1997). By combining the efficiency values calculated by our new approach with additional information derived from diverse research approaches (e.g., physiological studies, enzyme assays and genome sequencing), our analysis provides new insights in this discussion. Specifically, it identifies the importance of rDSR and of the TEA reduction step and questions the role of the APS pathway.

#### Role of rdsr

As noticed by Kelly (1982), facultative anaerobic SOB appear to generally have a higher energy conservation efficiency than obligate aerobic SOB, a trend clearly supported also by our results (Figure 2). This pattern can be understood by noticing that most facultative anaerobic thiosulfate oxidizing SOB are equipped with the “branched” pathway for thiosulfate oxidation (red arrows in Figure 1; Table 1), which is characterized by a truncated Sox system. In SOB that possess a Sox system that is not truncated (green arrows in Figure 1) a thiosulfate-oxidizing multi-enzyme (TOMES) complex catalyzes the complete oxidation of thiosulfate to sulfate, whereby the oxidation of the intermediate zero-valent sulfur is catalyzed by the soxCD enzymes of this complex (e.g., Ghosh and Dam, 2009). However, these enzymes are missing in the branched pathway and, instead, the transiently stored zero-valent sulfur is oxidized, after its reactivation to glutathione persulfide (GSSH), by other enzymes such as rDSR or the oxygen-dependent sulfur dioxygenase (SDO) (Rohwerder and Sand, 2003). While the complete TOMES complex directly channels all electrons derived from thiosulfate oxidation into the electron transport chain at the level of cyt *c* (Kelly et al., 1997), rDSR catalyzes the cytoplasmic oxidation of GSSH to sulfite and donates the 6 electrons into the electron transport chain eventually at the level of quinone (Holkenbrink et al., 2011). This rDSR-mediated mechanism might therefore provide more ATP and thus eventually lead to a higher energy conservation efficiency as compared to the Sox pathway.

rDSR is suspected to be the most ancient enzyme equipment of SOB and was probably employed by anaerobic anoxygenic phototrophs already in the early Archean era (Meyer and Kuever, 2007). The fact that rDSR is only conserved in extant facultative anaerobic SOB (and phototrophs) might therefore explain the apparent link between the high energy conservation efficiency and the capability of an anaerobic life style.

#### Role of the terminal electron acceptor reduction step

The importance of the type of TEA reductase for the energy conservation efficiency is suggested by our data compiled for the denitrifying SOB (Table 1). Namely, *Sulfurimonas denitrificans* exclusively relies on a periplasmically oriented nitrate reductase system (Nap) and does not possess a membrane-bound cytoplasmically oriented nitrate reductase system (Nar) (Sievert et al., 2008). Nitrate oxidation via Nap was shown not to contribute to the generation of proton-motive-force and thus ATP (Stewart et al., 2002), which is consistent with the rather poor energy conservation efficiency of this Epsilonproteobacterium. Most other known important denitrifying SOB, such as the large marine *Beggiatoa* and *Thioploca*, are equipped with a membrane-bound nitrate reductase system (Nar) (e.g., Crossman, 2007), which oxidizes nitrate on the cytoplasmic side of the membrane and thus contributes to the generation of proton motive force (Simon et al., 2008; Simon and Klotz, 2013). This is consistent with their generally higher energy conservation efficiency (Table 1). Thus, in denitrifying SOB the enzymes directly involved in TEA (NO^−^_3_) reduction appear to have at least a similar impact on the energy conservation efficiency as the sulfur oxidation pathway.

#### Role of the aps pathway

The role of the pathway of sulfite oxidation to sulfate, where the sulfite is derived, e.g., from sulfide oxidation by rDSR (Figure 1), has been extensively discussed in the past (e.g., Kelly, 2003). This oxidation step can be mediated either by sulfite:cyt *c*:oxidoreductase (SOAR) or via the APS pathway (Figure 1). Depending on the pathway of sulfur oxidation, SOARs are either associated with the TOMES complex or are complex-independent (Figure 1). While SOARs have been identified in all sulfite oxidizing free-living SOB, the APS pathway appears to be an “extra” pathway that is not crucial for the operation of complete sulfur oxidation (Kappler and Dahl, 2001). It is known that the APS pathway allows additional ATP gain via substrate level phosphorylation before the electrons enter the electron transport chain (Aminuddin, 1980), and its use should thus lead to a higher overall energy conservation efficiency. However, we did not identify a clear link between the efficiency and the presence of the APS pathway in our present data (Table 1), suggesting that the electron transport processes are more important than this substrate level phosphorylation step.

Nevertheless, the presence of the APS pathway might still be the cause of variations in efficiency among closely related species, such as *Beggiatoa* spp., as suggested by Nelson and Hagen (1995) and Hagen and Nelson (1997). Also, it has to be considered that the APS pathway in the anoxygenic phototroph *Allochromatium vinosum* only contributes to energy generation at high irradiances (Sánchez et al., 2001), which correspond to saturating availability of TEA and sulfur compound in chemolithotrophic SOB when sulfite oxidation via SOR might become the rate limiting step. Thus, also in SOB the APS pathway might operate only under certain growth conditions. It was not shown for all SOB listed in Table 1 that the APS pathway was active during the rate measurements, which might disguise a possible link between the calculated efficiency and the APS pathway.

### Sulfur oxidation at a variable sulfate-to-zero-valent-sulfur product ratio

An interesting outcome of our analysis was the way to calculate the stoichiometry of autotrophic sulfur oxidation at a variable S^0^:SO^2−^_4_ production ratio from the rates of only two reactants involved (see Section Stoichiometry from the efficiency of energy conservation and limited rate measurements). This calculation relied on an additional information about the activity of the SOB, namely on the relationship between the S^0^:SO^2−^_4_ production ratio (parameter *x*) and the energy conservation efficiency or the CO_2_:TEA consumption ratio. Ideally, this relationship should be determined experimentally; however, since such data are presently not available for an isolated SOB, our calculation assumed two specific forms of this relationship: a constant or a linearly decreasing or increasing function of *x*. In the following we clarify the biochemical interpretation and limitations of these assumptions.

The assumption of a constant CO_2_:TEA consumption ratio is equivalent to the assumption that the amount of ATP generated per electron transported to the TEA and the amount of ATP consumed per electron transported to the electron carrier (e.g., NAD^+^) are independent of the origin of the electrons, i.e., whether the electrons are derived from the first (S^0^ production) or second (S^0^ to SO^2−^_4_) oxidation step. This is because the rate of TEA reduction linearly correlates with the rate of electrons transported (e.g., 4 electrons per O_2_). In contrast, the assumption of a constant energy conservation efficiency implicitly assumes that both the first (reduced sulfur compound to S^0^) and second (S^0^ to SO^2−^_4_) oxidation steps have the same efficiency, i.e., the amount of ATP gained from TEA reduction and required for the electron carrier reduction per kJ of the calculated Gibbs free energy is the same for both oxidation steps.

The assumption of a linear dependency of the CO_2_:TEA consumption ratio on *x* considers that the amount of ATP generated during electron transport to the TEA and the amount of ATP required during electron transport to the electron carriers depend on the origin of the electrons. Since the transported electrons derived from each oxidation reaction will have a specific constant potential to generate ATP, a linear dependency of the CO_2_:TEA ratio on *x* will result from the linear “mixing” of the electrons originating from the first and second oxidation step. Similarly, the assumption of a linear dependency of the efficiency on *x* is a consequence of the linear “mixing” of the efficiencies specific for the complete and incomplete sulfur oxidation reactions.

It should be noted that a major shortcoming of the CO_2_:TEA-based approach is that it does not consider the experimental conditions, such as reactant concentrations or the effect of the oxygen concentration on the efficiency of the CO_2_ fixation pathway (ε_CO2_). These conditions are expected to affect the ATP yield and ATP consumption per transported electron. In contrast, the efficiency-based approach does allow to account for this. Specifically, if the conditions were well-constrained during the experimental determination of the relation between the efficiency and *x*, and during the independent measurement of two reactant rates, our framework would make it possible, at least theoretically, to precisely predict the complete stoichiometry of autotrophic sulfur oxidation, and thus primary productivity, at any experimental condition.

### Interpretation of reactant fluxes in the environment

In mixed microbial communities different processes that utilize the same reactants often spatially overlap, which hampers our ability to interpret the measured net consumption/production rates of the reactants involved. To illustrate how our framework can aid such interpretation, we consider microbial mats inhabited by one dominant SOB in addition to other bacteria. In such mats, it is often observed that, in the zone where sulfide is oxidized with oxygen, the ratio between the steady-state O_2_ and ΣH_2_S consumption rates is 2 (e.g., Jørgensen et al., 2010). What does this ratio suggest about the S, O, and C cycling in the system?

Knowing that under steady-state conditions SOB exclusively produce SO^2−^_4_ and no S^0^ (Nelson et al., 1986), the O_2_:ΣH_2_S ratio of 2 suggests complete aerobic sulfide oxidation to sulfate (e.g., Jørgensen et al., 2010). Although this interpretation is correct, it ignores carbon cycling within the sulfide oxidation zone. If carbon cycling is taken into account, as it should be because the SOB that mediate the aerobic sulfide oxidation must also grow, this interpretation must be reevaluated.

Our framework implies that the general stoichiometry of aerobic sulfide oxidation coupled to CO_2_ fixation under steady state is (Table S1A)

$$\begin{array}{l} {\text{H}}_{2}\text{S}+\text{2y }{\text{O}}_{2}+2(1-y)\;{\text{CO}}_{2}+2(1-y)\;{\text{H}}_{2}\text{O}\to \\ {\text{SO}}_{4}^{2-}+2(1-y)\;{\text{CH}}_{2}\text{O}+2{\text{H}}^{+}. \end{array}$$

Thus, the O_2_:ΣH_2_S ratio due to the SOB activity is 2*y*, which is generally lower than 2 because 0<*y*<1 (Table S2). This means that in the mixed community part of the observed O_2_ consumption must be due to another kind of activity. A plausible candidate is aerobic respiration according to the stoichiometry

$$\begin{array}{l} 2(1-y)\;{\text{O}}_{2}+2(1-y)\;{\text{CH}}_{2}\text{O}\to \\ 2(1-y)\;{\text{CO}}_{2}+2(1-y)\;{\text{H}}_{2}\text{O}. \end{array}$$

This shows that when the two processes occur simultaneously the O_2_:ΣH_2_S ratio of 2 can be achieved only if the stoichiometric coefficient for CO_2_ in the SOB-driven reaction is the same as that in the aerobic respiration reaction [i.e., 2(1−*y*)]. Thus, assuming that the steady-state conditions are met, the O_2_:ΣH_2_S ratio of 2 suggests that the biomass built up by the dominant SOB through aerobic sulfide oxidation is completely recycled within the sulfide oxidation zone via aerobic respiration. Note that this argument is independent of *y* and thus of the energy conservation efficiency of the dominant SOB. The efficiency only determines the fraction of the O_2_ flux that is used for organic carbon recycling.

This analysis can be extended to cases when the O_2_:ΣH_2_S consumption ratio in the sulfide oxidation zone is different from 2 while the stoichiometry of the SOB activity remains unchanged. Specifically, O_2_:ΣH_2_S<2 would suggest export of the SOB-generated biomass out of the sulfide oxidation zone (e.g., for degradation by anaerobic processes), whereas O_2_:ΣH_2_S>2 would indicate import of an external reductant (e.g., organic carbon) into, and its aerobic oxidation within, the zone.

### Implications for niche differentiation among SOB

Past and present research on SOB revealed that (i) sulfur oxidation pathways are highly diverse and characterized by a degree of redundancy (Figure 1), (ii) the product of sulfide oxidation can vary substantially (between S^0^ and SO^2−^_4_) even in one SOB dependent on growth conditions and phase, (iii) the energy conservation efficiency of SOB varies widely (Table 1), and (iv) SOB possessing one of the most ancestral enzymes involved in sulfur oxidation (rDSR) appear to be also the most efficient (Table 1). In the following we attempt a synthesis of these findings in the context of niche differentiation among SOB.

To understand the apparent diversification toward lower energy conservation efficiency, it needs to be realized that efficiency is generally not the only measure of success in a given environment. Generally, the success is determined by the growth rate, which is a *product* of the substrate uptake rate and the growth yield (i.e., the amount of organic carbon synthesized per unit of substrate utilized). Since the growth yield and efficiency are directly related (as follows from Table S1 and Equations 8 and 30), being efficient is only one of the possibilities for being successful. If organisms compensate their inefficient energy conservation by speed with which they utilize the substrate, they can be equally or even more successful than the efficient ones (e.g., Sorokin and Kuenen, 2005). These strategies are, however, likely dominant only in environments with unlimited substrates.

The situation is different in environments where the substrates are limited, e.g., because of a limited external supply. In the context of SOB this can occur if the flux of the reduced sulfur compound (S_red_) or of the TEA into the sulfur oxidizing zone is capped, e.g., by the rate of diffusion or by the substrate concentration in the seeping water delivering the substrate through advection. Under such circumstances, the flux of the external substrate supply will define the maximum substrate uptake rate. Ignoring the role of other phenotypes such as motility, this implies that the success of a specific SOB in the sulfur oxidizing zone will depend only on its energy utilization efficiency.

A possible strategy for an SOB to become successful under such circumstances is to match its substrate uptake stoichiometry to the TEA:S_red_ ratio fixed by the environment. Such an SOB will utilize the available substrates completely, leaving nothing for potential competitors. Table S1 and Equation (30) show that this can be achieved essentially by “adjusting” two parameters: *y*, and thus the energy conservation efficiency, and *x*, i.e., the S^0^:SO^2−^_4_ product ratio. Which of the parameters is “adjusted” depends on the time-scale at which the environmental setting (the TEA:S_red_ ratio) varies.

Specifically, a long-term stable environment with a low TEA:S_red_ ratio should select for an SOB with a high efficiency, and vice versa. This could, for example, provide a possible explanation why SOB living in symbiotic association with invertebrates are highly efficient (Table 1). Specifically, symbiotic SOB need to compete with the host for O_2_ and thus likely face O_2_ limitation, which requires them to have evolved highly efficient substrate utilization. On the other hand, they can “afford” to be highly efficient because they do not have to retain flexibility in the presumably stable environment regulated by the host.

In contrast, environments with short-term TEA:S_red_ fluctuations should select for SOB that possess mechanisms for rapid optimization of the substrate utilization stoichiometry. Phenotypic traits operating on short time-scales, such as motility coupled to chemotaxis or the ability to store TEA (nitrate), will likely play a role. However, another important mechanism could be the ability to perform sulfur oxidation at a variable S^0^: SO^2−^_4_ ratio. Our framework formulates this ability through the parameter *x* while the overall stoichiometry of the autotrophic sulfur oxidation reaction is additionally dependent on the parameter *y*. This makes it possible to predict the optimal range of sulfur oxidation efficiencies that an SOB should have to be successful in a given fluctuating environment. Specifically, this range should be such that the corresponding TEA:S_red_ utilization ratios during the incomplete (at *x* = 1) and complete (at *x* = 0) oxidation of S_red_ would match the range of the TEA:S_red_ fluctuations imposed by the environment.

To illustrate this, we assume that the O_2_:ΣH_2_S flux ratio available in the environment rapidly fluctuates between 0.45 and 1.7. To optimally utilize the available O_2_ and total sulfide (ΣH_2_S) pool at all times, the SOB should vary the product of its sulfide oxidation between two extremes: S^0^ when the O_2_:ΣH_2_S ratio reaches 0.45, and SO^2−^_4_ when the O_2_:ΣH_2_S ratio reaches 1.7. As follows from Table S1, the O_2_:ΣH_2_S ratio is equal to *y*(2-1.5*x*), which yields *y* = 0.9 to satisfy the condition O_2_:ΣH_2_S = 0.45 at *x* = 1 (incomplete sulfide oxidation) and *y* = 0.85 to satisfy the condition O_2_:ΣH_2_S = 1.7 at *x* = 0 (complete sulfide oxidation). Assuming standard conditions and Calvin cycle, the efficiency of the most successful SOB should therefore vary between ε_II_ = 4.3% and ε_II_ = 8% for the incomplete and complete aerobic sulfide oxidation, respectively, with the corresponding stoichiometries varying between

$${\text{H}}_{2}\text{S}+0.45\;{\text{O}}_{2}+0.05\;{\text{CO}}_{2}\to {\text{S}}^{0}+0.05\;{\text{CH}}_{2}\text{O}+0.95\;{\text{H}}_{2}\text{O}$$

and

$$\begin{array}{l} {\text{H}}_{2}\text{S}+1.7\;{\text{O}}_{2}+0.3\;{\text{CO}}_{2}+0.3\;{\text{H}}_{2}\text{O}\to \\ {\text{SO}}_{4}^{2-}+0.3\;{\text{CH}}_{2}\text{O}+2\;{\text{H}}^{+}. \end{array}$$

There are possibly other strategies that SOB living in fluctuating environments employ to become successful, including minimization of S^0^ production to prevent cell bursting (if S^0^ is stored intra-cellularly) or maximization of the growth yield. Although they can be explored within our theoretical framework, their thorough theoretical analysis would go beyond the scope of this study. Nevertheless, the examples and analyses discussed above demonstrate that the concepts and equations put forward in this study provide a generic theoretical framework that can help researchers gain new insights into the activity of sulfur oxidizing bacteria in a wide range of environments and their niches.

### Conflict of interest statement

The authors declare that the research was conducted in the absence of any commercial or financial relationships that could be construed as a potential conflict of interest.
