# Supplementary material for: Assessment of the stoichiometry and efficiency of CO2 fixation coupled to reduced sulfur oxidation
Source: Front Microbiol. 2015 May 21;6:484. doi: 10.3389/fmicb.2015.00484 (PMC4440400; doi:10.3389/fmicb.2015.00484)
Supplement: Supplementary file 1 [file Presentation1.PDF]

Supplementary Material

Assessment of the stoichiometry and efficiency of CO_2 fixation coupled to reduced sulfur oxidation

Judith M. Klatt[1*], Lubos Polerecky[1,2*]

[1] Max Planck Institute for Marine Microbiology, Bremen, Germany
[2]Department of Earth Sciences – Geochemistry, Faculty of Geosciences, Utrecht University, Utrecht, The Netherlands

***Correspondence:**
Judith M. Klatt, Max Planck Institute for Marine Microbiology, Celsiusstr. 1, D-28357 Bremen, Germany
jklatt@mpi-bremen.de
Lubos Polerecky, Department of Earth Sciences – Geochemistry, Faculty of Geosciences, Utrecht University, Princetonplein 9, 3584 CC, Utrecht, The Netherlands
l.polerecky@uu.nl

Content

1. Implementation of the efficiency calculation in R
2. Calculation of the stoichiometry of autotrophic sulfur oxidation – other examples
    1.1. *Beggiatoa* strain MS-81-1c
    1.2. *Thioploca* bundles
3. Net and gross fluxes of O_2 and CO_2 in *Riftia pachyptila* tubeworms
4. Details of efficiency calculations for *Sulfurimonas denitrificans* and *Thioalkalivibrio versutus*
5. Supplementary Figures
    5.1. Figure S1: Schematic representation of the biochemical transformations involved in energy conservation by SOB.
    5.2. Figure S2: Sensitivity analysis of the calculated energy conservation efficiency of sulfur oxidation coupled to CO_2 fixation.
6. Supplementary Table S1
    6.1. Table S1A: Generalized mass-balanced equations for aerobic oxidation of reduced sulfur species coupled to CO_2 fixation.
    6.2. Table S1B: Generalized mass-balanced equations for anaerobic oxidation of reduced sulfur species coupled to DNRA and CO_2 fixation
    6.3. Table S1C: Generalized mass-balanced equations for anaerobic oxidation of reduced sulfur species coupled to denitrification and CO_2 fixation
    6.4. Table S1D: Generalized mass-balanced equations for anaerobic oxidation of reduced sulfur species coupled to nitrite reduction and CO_2 fixation.
    6.5. Table S1E: Generalized mass-balanced equation for anaerobic oxidation of reduced sulfur species coupled to N_2O reduction and CO_2 fixation.
7. Supplementary Table S2: Stoichiometries of autotrophic sulfur oxidation calculated for specific SOB strains based on experimental data available in the literature.
8. Supplementary References

1. Implementation of the efficiency calculation in R

R-scripts for the calculation of the energy conservation efficiency of autotrophic sulfur oxidation processes can be downloaded from http://nanosims.geo.uu.nl/SOX. The required functions are loaded in R using command

```
source('SOX_functions.R')
```

To calculate the efficiency for a given stoichiometry, the values of x and y need to be provided as input parameters. In the following we assume that these values are equal to $x = 0$ and $y = 0.825$. The efficiency of *aerobic* sulfide oxidation at standard biochemical conditions is calculated with the traditional approach using command

```
aerob_efficiency(x=0,y=0.825,Sred="H2S",approach=1)$eff
```

To calculate the efficiency using our new approach one needs to additionally specify the carbon fixation pathway, e.g.,

```
aerob_efficiency(x=0,y=0.825,Sred="H2S",approach=2,Cfix="Calvin")$eff
```

To calculate the efficiency of aerobic thiosulfate oxidation, one needs to specify thiosulfate as the reduced sulfur compound, i.e.,

```
aerob_efficiency(x=0,y=0.825,Sred="S2O3",approach=2,Cfix="Calvin")$eff
```

By default, the efficiency is calculated at standard biochemical conditions. To calculate it at different conditions, one needs to specify the concentrations of the reactants involved. For example, for the concentrations of total sulfide $[\sum H_2S] = 30$ µM, $[SO_4^{2-}] = 20$ mM, $[O_2] = 100$ µM, total inorganic carbon $[CO_2] = 2.5$ mM, $[NAD^+] = [NADH] = 1$ mM, pH of 6 and temperature of 15 °C, the efficiency is calculated using command

```
aerob_efficiency(x=0,y=0.825,Sred="H2S",approach=2,Cfix="Calvin",S=30e-6,
sulfate=20e-3,O2=100e-6,CO2=2.5e-3,NAD=1e-3,NADH=1e-3,pH=6, Temp=15)$eff
```

The efficiency of anaerobic sulfur oxidation coupled to denitrification is calculated using the function `denit_efficiency`. The input parameters are the same as for the `aerob_efficiency` function. For example, to calculate the efficiency for an SOB that performs thiosulfate oxidation coupled to denitrification and employs the rTCA cycle as the CO_2 fixation pathway using our new approach, one needs to use command

```
denit_efficiency(x=0,y=0.825,Sred="S2O3",approach=2,Cfix="rTCA")$eff
```

By default, the efficiency is calculated at standard biochemical conditions. To calculate the efficiency at different conditions, one needs to specify the concentrations of the reactants involved. For example, for the concentrations $[S_2O_3^{2-}] = 30$ µM, $[SO_4^{2-}] = 20$ mM, $[NO_3^-] = 100$ µM, total inorganic carbon $[CO_2] = 2.5$ mM, $[N_2] = 10$ mM, $[NAD^+] = [NADH] = [Fd_{ox}] = [Fd_{red}] = [FAD] = [FADH_2] = 1$ mM, pH of 6 and temperature of 15 °C, the efficiency is calculated using command

```
denit_efficiency(x=0,y=0.825,Sred="S2O3",approach=2,Cfix="rTCA",S=30e-6,
sulfate=20e-3,NO3=100e-6,N2=10e-3,CO2=2.5e-3,NAD=1e-3,NADH=1e-3,Fdox=1e-3,
Fdred=1e-3,FAD=1e-3,FADH2=1e-3,pH=6, Temp=15)$eff
```

If `$eff` in the above function calls is omitted, the function call will additionally display the values of the Gibbs free energy for the energy generating and energy conserving reactions and of the quotients Q. The values of the partial efficiencies ($\varepsilon_{SO,II,min}$,, ε_{CO2} and $\varepsilon_{RET,II,min}$) are displayed only for our new calculation approach (i.e., when `approach=2`)

The website also contains additional examples illustrating (i) the calculation of sulfur oxidation stoichiometry from a given efficiency and the ratio between the reduced sulfur compound and the terminal electron acceptor (TEA) (file `stoichiometry_examples.R`), (ii) the sensitivity analysis of the efficiency calculation (file `sensitivity_analysis.R`; examples shown in Fig. S2), and (iii) the dependence of the CO_2:TEA ratio on the parameter *x* (file `CO2_TEA_x.R`; examples shown in Fig. 3).

2. Calculation of the stoichiometry of autotrophic sulfur oxidation – other examples

1.1. *Beggiatoa* strain MS-81-1c

Hagen & Nelson (1997) found that CO_2 fixation in the *Beggiatoa* strain MS-81-1c occurred at a CO_2:O_2 consumption ratio of 0.596 and was accompanied by a considerable production of S^0 (S^0:O_2 ratio of 0.04). According to Table S1A, this can be transformed into equations $\nu_{CO2}/\nu_{O2} = (1–y)/y = 0.596$ and $\nu_{S0}/\nu_{O2} = x/[y(2\text{-}1.5x)] = 0.04$, which yield $y = 0.64$ and $x = 0.05$. Thus, in this strain, aerobic oxidation of sulfide is performed according to equation

$$H_2S + 1.23\ O_2 + 0.69\ CO_2 + 0.64\ H_2O \rightarrow 0.95\ SO_4^{2-} + 0.05\ S^0 + 0.69\ CH_2O + 1.9\ H^+$$

with 64% of the sulfide pool used in the energy gaining reaction with oxygen, 5% of the sulfide pool converted to zero-valent sulfur and the remaining 95% completely oxidized to SO_4^{2-}.

1.2. *Thioploca* bundles

Otte et al. (1999) found that intact bundles of *Thioploca* sp. fixed CO_2 in the absence of externally supplied sulfide by oxidizing their intra-cellularly stored zero-valent sulfur to sulfate accompanied by nitrate reduction to ammonium. When corrected for the rates in disrupted control samples, the production of NH_4^+ and SO_4^{2-} occurred at a ratio of $\nu_{NH4}/\nu_{SO4} = 0.5$. This ratio is equal to 0.75*y*, which yields $y = 0.67$. Thus, zero-valent sulfur oxidation in the *Thioploca* bundles coupled to ammonification and CO_2 fixation is expected to occur according to equation

$$S^0 + 0.5\ NO_3^- + 0.5\ CO_2 + 2\ H_2O \rightarrow SO_4^{2-} + 0.5\ NH_4^+ + 0.5\ CH_2O + 1\ H^+,$$

with 67% of the zero-valent sulfur pool used in the energy gaining reaction with nitrate.

Based on this stoichiometry, a growth yield of 0.5 mole of CO_2 per mole of S^0 and a NO_3^-:CO_2 consumption ratio of 1 are expected. However, in another experiment with *Thioploca* bundles Otte et al. (1999) measured a NO_3^-:CO_2 consumption ratio of about 0.55. If this ratio is used as a starting value, Table S1B implies that $\nu_{NO3}/\nu_{CO2} = 0.75y/[1.5(1–y)] = 0.55$, which yields $y = 0.8$ and thus a considerably different stoichiometry of the sulfur oxidation reaction:

$$S^0 + 0.6\ NO_3^- + 0.3\ CO_2 + 1.9\ H_2O \rightarrow SO_4^{2-} + 0.6\ NH_4^+ + 0.3\ CH_2O + 0.8\ H^+.$$

This apparent inconsistency suggests that during the different experiments reported by Otte et al. (1999) the studied *Thioploca* bundles (i) had a different physiological status, (ii) that an unidentified additional electron donor or acceptor was involved, or (iii) that significant sulfur cycling within the bundles occurred. The latter possibility would be consistent with the finding of filamentous sulfate reducers co-inhabiting the *Thioploca* sheaths (Fukui et al., 1999; Teske et al., 2009).

3. Net and gross fluxes of O_2 and CO_2 in *Riftia pachyptila* tubeworms

Girguis et al. (2002) performed several high-pressure respirometry experiments to determine net fluxes of sulfide, oxygen and CO_2 for the host-symbiont system of tubeworms *Riftia pachyptila*. Specifically, for the untreated tubeworms they observed a net O_2 consumption rate of

~10 µmol O_2 g^{-1} h^{-1}, while after the addition of amiloride the O_2 consumption rate decreased to ~2.5 µmol O_2 g^{-1} h^{-1} (both values estimated from Figure 5 in Girguis et al., 2002). Additionally, the treatment with amiloride resulted in a net production of CO_2, suggesting that the symbiont cells became inactive. Assuming that the amiloride treatment inhibited completely the activity of the symbionts but had no effect on the host, these values suggest that host respiration contributes an estimated 25% to the net O_2 consumption of the whole-worm symbiosis while the symbionts-mediated autotrophic sulfide oxidation activity contributes the remaining 75%.

To calculate the gross rate of CO_2 fixation by the symbionts from the net rate of CO_2 consumption by the whole-worm symbiosis, it is necessary to know the rate of CO_2 production due to the respiration by the host. We estimated this rate by assuming that the host consumes O_2 and produces CO_2 at a ratio of 1:1. Thus, using the values reported by Girguis et al. (2002) for an experiment where they simultaneously measured the net rates of O_2 consumption (12.4 µmol O_2 g^{-1} h^{-1}), CO_2 consumption (12.45 µmol CO_2 g^{-1} h^{-1}) and sulfide consumption (6.75 µmol $\sum H_2S$ g^{-1} h^{-1}) for the whole-worm symbiosis, we estimated the gross O_2 consumption rate by the symbionts to be $0.75 \times 12.4 = 9.3$ µmol O_2 g^{-1} h^{-1} and the corresponding gross rate of CO_2 fixation to be $12.45 + 0.25 \times 12.4 = 15.55$ µmol CO_2 g^{-1} h^{-1}.

4. Details of efficiency calculations for *Sulfurimonas denitrificans* and *Thioalkalivibrio versutus*

Hoor (1981) reported that *Sulfurimonas denitrificans* performs aerobic thiosulfate oxidation coupled to CO_2 fixation with a growth yield of 0.333 mole of carbon per mole of thiosulfate, with thiosulfate completely oxidised to sulfate. Using Table S1A, this information leads to equations $\nu_{CO2}/\nu_{S2O3} = (1–y)(2–1.5x) = 0.333$ and $x = 0$, which yield $y = 0.833$ and the corresponding equation

$$S_2O_3^{2-} + 1.67\ O_2 + 0.33\ CO_2 + 1.33\ H_2O \rightarrow 2\ SO_4^{2-} + 0.33\ CH_2O + 2\ H^+.$$

Using these stoichiometric coefficients and the ΔG_f^0 values tabulated by Thauer (1977), and considering that *Sulfurimonas denitrificans* employs the rTCA cycle, we obtained $\Delta G_r^0(O_2\ \text{red}) = -842.29$ kJ mol^{-1}, $\Delta G_r^0(CO_2\ \text{red}) = 131.77$ kJ mol^{-1}, $[\Delta G_r^0(CO_2\ \text{fix}) + \Delta G_r^0(\text{RET})] = 335.52$ kJ mol^{-1} and $[\Delta E_r(CO_2\ \text{fix}) + \Delta G_r^0(\text{RET})] = 343.52$ kJ mol^{-1}. Assuming standard biochemical conditions, substitution of these values together with the value of y to Eqs. 8, 30 and 37 gave the efficiencies $\varepsilon_I = 0.0314$, $\varepsilon_{II} = 0.0799$ and $\varepsilon_{SO,II,min} = 0.0818$.

Sorokin et al. (2001) reported that *Thioalkalivibrio versutus* performs aerobic thiosulfate oxidation coupled to CO_2 fixation with a growth yield of 5 g protein per mole of thiosulfate, with thiosulfate completely oxidised to sulfate. Assuming a carbon:protein ratio of 0.75 (Hoor, 1981; Simon and Azam, 1989), this leads to equations $\nu_{CO2}/\nu_{S2O3} = (1–y)(2–1.5x) = 0.3125$ and x=0, which yield y=0.859 and the corresponding equation

$$S_2O_3^{2-} + 1.69\ O_2 + 0.31\ CO_2 + 1.31\ H_2O \rightarrow 2\ SO_4^{2-} + 0.31\ CH_2O + 2\ H^+.$$

Using these stoichiometric coefficients and the ΔG_f^0 values tabulated by Thauer (1977), and considering that *Thioalkalivibrio versutus* employs the Calvin cycle, we obtained $\Delta G_r^0(O_2\ \text{red}) = -842.29$ kJ mol^{-1}, $\Delta G_r^0(CO_2\ \text{red}) = 131.77$ kJ mol^{-1}, $[\Delta G_r^0(CO_2\ \text{fix}) + \Delta G_r^0(\text{RET})] = 362.70$ kJ mol^{-1} and $[\Delta E_r(CO_2\ \text{fix}) + \Delta G_r^0(\text{RET})] = 469.36$ kJ mol^{-1}. At standard biochemical conditions this yields the efficiency values of $\varepsilon_I = 0.0289$, $\varepsilon_{II} = 0.0796$ and $\varepsilon_{SO,II,min} = 0.1030$.

5. Supplementary Figures

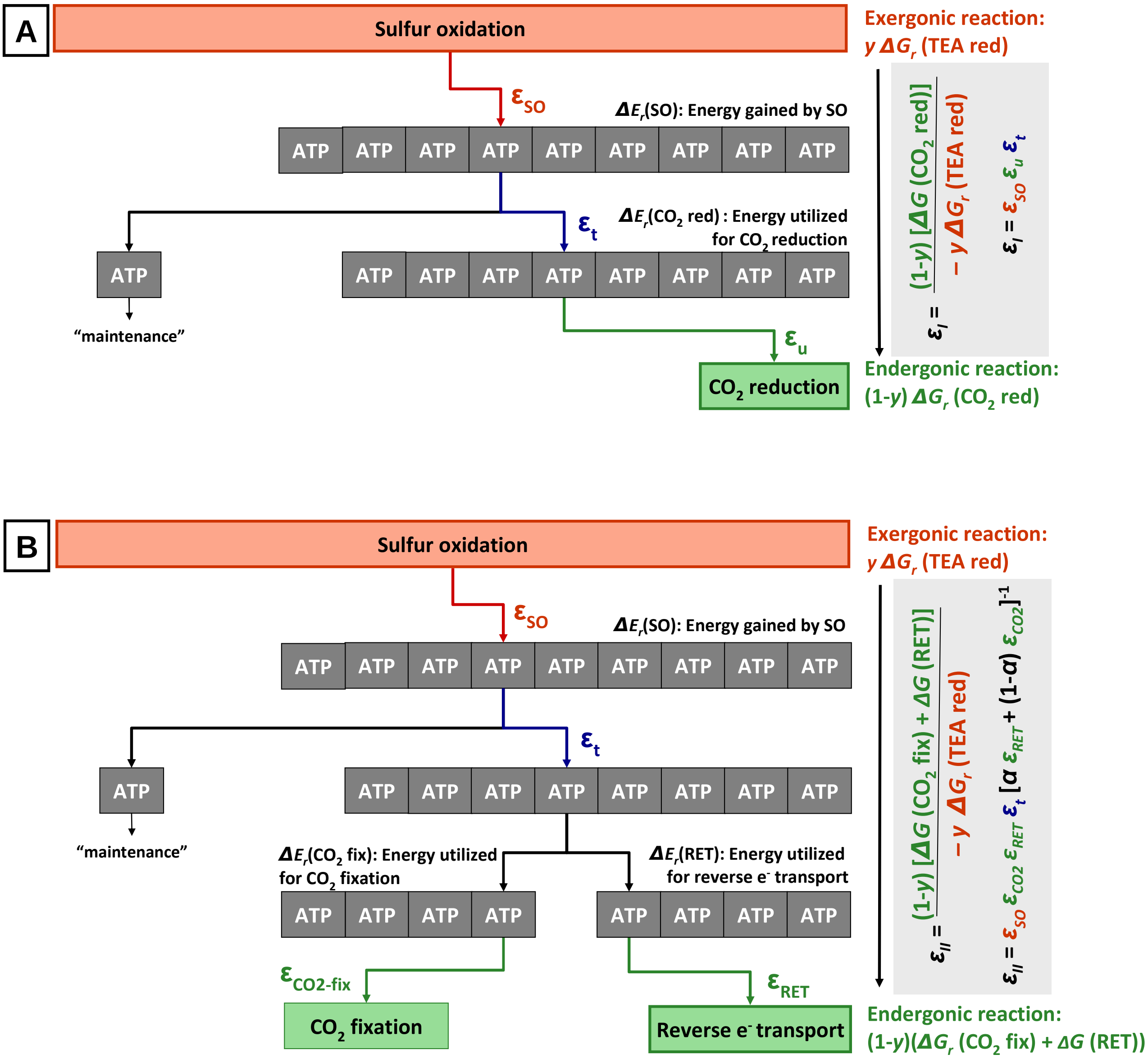

Figure S1: Schematic representation of the biochemical transformations involved in energy conservation by SOB. Panels A and B correspond to the traditional and our new approach for calculating the energy conservation efficiency, respectively. In both approaches energy available from sulfur oxidation (SO) with the terminal electron acceptor (TEA) is transformed into the biochemical currency ATP with an efficiency ε_{SO}. A fraction ε_t of this ATP is utilized for CO_2 reduction, while the remaining fraction $(1 - \varepsilon_t)$ is used for cellular maintenance reactions. In the traditional approach the ATP is utilized for CO_2 reduction with an efficiency ε_u. In contrast, our new approach considers that ATP is actually driving two processes: reverse electron transport to reduce electron carriers (e.g., NAD^+, FAD, Ferredoxin), and the CO_2 reduction reactions in the respective CO_2 fixation pathway. The corresponding efficiencies of these processes are ε_{RET} and ε_{CO2}. ΔE_r and ΔG_r correspond to the energy of the reaction in the form of ATP and the Gibbs free energy of the reaction, respectively.

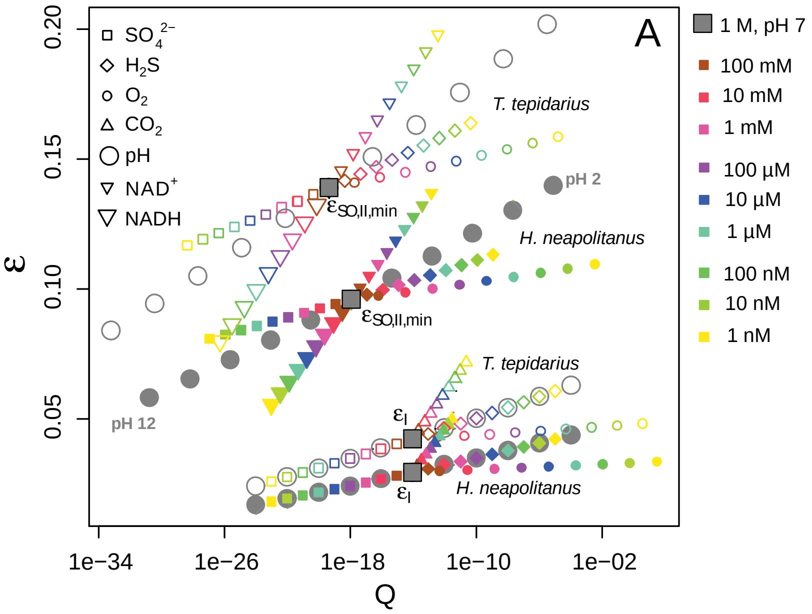

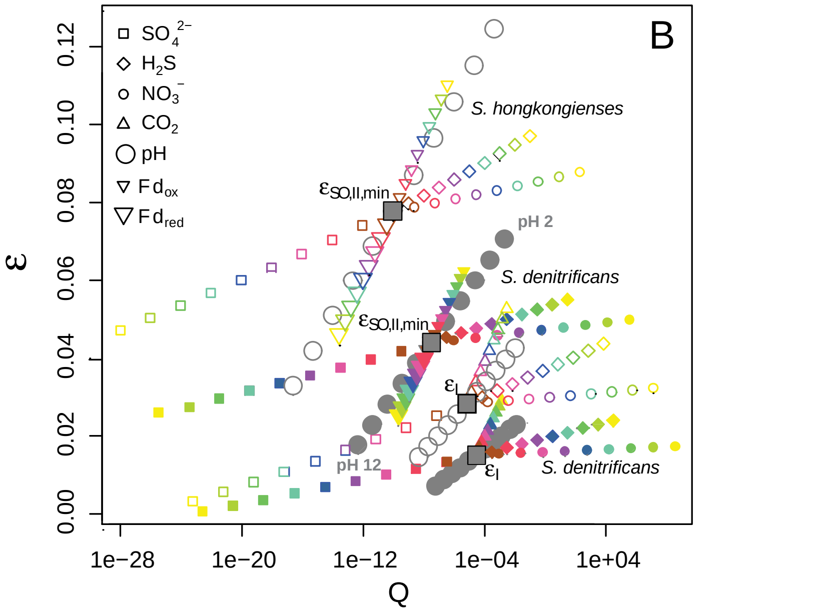

Figure S2: Sensitivity analysis of the calculated energy conservation efficiency of sulfur oxidation coupled to CO_2 fixation. Shown are values of ε_I and $\varepsilon_{SO,II,min}$ calculated for **(A)** aerobic sulfide oxidizing bacteria *Thermithiobacillus tepidarius* and *Halothiobacillus neapolitanus* and for **(B)** denitrifying thiosulfate oxidizing bacteria *Sulfurimonas hongkongensis* and *Sulfurimonas denitrificans*.. In all calculations we separately varied the concentrations of each reactant from 1M to 1 nM, or the pH from 2 to 12, while keeping the other reactant concentrations constant at 1 M or pH 7. For the denitrifying SOB, which employ the rTCA cycle for CO_2 fixation, the slope of the variation of $\varepsilon_{SO,II,min}$ with the concentration of the oxidized/reduced electron carriers NAD^+/NADH and FAD/$FADH_2$ is the same as that for the oxidized/reduced Ferredoxin (Fd_{ox}/Fd_{red}). The sensitivity of $\varepsilon_{SO,II,min}$ to changes in the concentrations of NAD^+/NADH and FAD/$FADH_2$ is lower than the sensitivity to changes in the concentrations of Fd_{ox}/Fd_{red}. Data-points corresponding to NAD^+/NADH and FAD/$FADH_2$ are, however, not displayed to maintain graph clarity. In all graphs the large gray-filled squares represent the efficiency calculated at standard biochemical conditions (1M concentrations of all reactants, pH=7, temperature 25 °C).

Table S1A: Generalized mass-balanced equations for aerobic oxidation of reduced sulfur species coupled to CO_2 fixation.

	$\sum H_2S$ oxidation	$S_2O_3^{2-}$ oxidation
Energy-generating reactions	$H_2S + 0.5\ O_2 \rightarrow S^0 + H_2O$	$S_2O_3^{2-} + 0.5\ O_2 \rightarrow S^0 + SO_4^{2-}$
	$H_2S + 2\ O_2 \rightarrow SO_4^{2-} + 2\ H^+$	$S_2O_3^{2-} + 2\ O_2 + H_2O \rightarrow 2\ SO_4^{2-} + 2\ H^+$
	$\mathbf{H_2S + (2–1.5\mathit{x})\ O_2 \rightarrow \mathit{x}\ S^0 + (1–\mathit{x})\ SO_4^{2-} + \mathit{x}\ H_2O + (2–2\mathit{x})\ H^+}$	$\mathbf{S_2O_3^{2-} + (2–1.5\mathit{x})\ O_2 + (1–\mathit{x})\ H_2O \rightarrow \mathit{x}\ S^0 + (2–\mathit{x})\ SO_4^{2-} + (2–2\mathit{x})\ H^+}$
Energy-conserving reactions (traditional approach)	$H_2S + 0.5\ CO_2 \rightarrow S^0 + 0.5\ CH_2O + 0.5\ H_2O$	$S_2O_3^{2-} + 0.5\ CO_2 + 0.5\ H_2O \rightarrow S^0 + SO_4^{2-} + 0.5\ CH_2O$
	$H_2S + 2\ CO_2 + 2\ H_2O \rightarrow SO_4^{2-} + 2\ CH_2O + 2\ H^+$	$S_2O_3^{2-} + 2\ CO_2 + 3\ H_2O \rightarrow 2\ SO_4^{2-} + 2\ CH_2O + 2\ H^+$
	$\mathbf{H_2S + (2–1.5\mathit{x})\ CO_2 + (2–2.5\mathit{x})\ H_2O \rightarrow \mathit{x}\ S^0 + (1–\mathit{x})\ SO_4^{2-} + (2–1.5\mathit{x})\ CH_2O + (2–2\mathit{x})\ H^+}$	$\mathbf{S_2O_3^{2-} + (2–1.5\mathit{x})\ CO_2 + (3–2.5\mathit{x})\ H_2O \rightarrow \mathit{x}\ S^0 + (2–\mathit{x})\ SO_4^{2-} + (2–1.5\mathit{x})\ CH_2O + (2–2\mathit{x})\ H^+}$
Energy-conserving reactions (Calvin cycle, new approach)[1]	$H_2S + NAD^+ \rightarrow S^0 + NADH + H^+$	$S_2O_3^{2-} + NAD^+ + H_2O + 2\ H^+ \rightarrow S^0 + SO_4^{2-} + 4\ NADH$
	$H_2S + 4\ NAD^+ + 4\ H_2O \rightarrow SO_4^{2-} + 4\ NADH + 6\ H^+$	$S_2O_3^{2-} + 4\ NAD^+ + 5\ H_2O \rightarrow 2\ SO_4^{2-} + 4\ NADH + 6\ H^+$
	$\mathbf{H_2S + (4–3\mathit{x})\ NAD^+ + (4–4\mathit{x})\ H_2O \rightarrow \mathit{x}\ S^0 + (1–\mathit{x})\ SO_4^{2-} + (4–3\mathit{x})\ NADH + (6–5\mathit{x})\ H^+}$	$\mathbf{S_2O_3^{2-} + (4–3\mathit{x})\ NAD^+ + (5–4\mathit{x})\ H_2O \rightarrow \mathit{x}\ S^0 + (2–\mathit{x})\ SO_4^{2-} + (4–3\mathit{x})\ NADH + (6–8\mathit{x})\ H^+}$
Energy-conserving reactions (rTCA cycle, new approach)[1]	$H_2S + 0.5\ NAD^+ + 0.67\ Fd_{ox} + 0.17\ FAD \rightarrow$ $S^0 + 0.5\ NADH + 0.67\ Fd_{red} + 0.17\ FADH_2 + 1.17\ H^+$	$S_2O_3^{2-} + 0.5\ NAD^+ + 0.67\ Fd_{ox} + 0.17\ FAD + H_2O \rightarrow$ $S^0 + SO_4^{2-} + 0.5\ NADH + 0.67\ Fd_{red} + 0.17\ FADH_2 + 1.17\ H^+$
	$H_2S + 2\ NAD^+ + 2.67\ Fd_{ox} + 0.67\ FAD + 4\ H_2O \rightarrow$ $SO_4^{2-} + 2\ NADH + 2.67\ Fd_{red} + 0.67\ FADH_2 + 6.67\ H^+$	$S_2O_3^{2-} + 2\ NAD^+ + 2.67\ Fd_{ox} + 0.67\ FAD + 5\ H_2O \rightarrow$ $2\ SO_4^{2-} + 2\ NADH + 2.67\ Fd_{red} + 0.67\ FADH_2 + 6.67\ H^+$
	$\mathbf{H_2S + (2 – 1.5\mathit{x})\ NAD^+ + (2.67 – 2\mathit{x})\ Fd_{ox} + (0.67 – 0.5\mathit{x})\ FAD + (4 – 4\mathit{x})\ H_2O \rightarrow}$ $\mathbf{\mathit{x}\ S^0 + (1 – \mathit{x})\ SO_4^{2-} + (2 – 1.5\mathit{x})\ NADH + (2.67 – 2\mathit{x})\ Fd_{red} + (0.67 – 0.5\mathit{x})\ FADH_2 + (6.67 – 5.5\mathit{x})\ H^+}$	$\mathbf{S_2O_3^{2-} + (2 – 1.5\mathit{x})\ NAD^+ + (2.67 – 2\mathit{x})\ Fd_{ox} + (0.67 – 0.5\mathit{x})\ FAD^+ + (5 – 4\mathit{x})\ H_2O \rightarrow}$ $\mathbf{\mathit{x}\ S^0 + (1 – \mathit{x})\ SO_4^{2-} + (2 – 1.5\mathit{x})\ NADH + (2.67 – 2\mathit{x})\ Fd_{red} + (0.67 – 0.5\mathit{x})\ FADH + (6.67 – 5.5\mathit{x})\ H^+}$
Generalized coupled reaction	$\mathbf{H_2S + \nu_{O2}\ O_2 + \nu_{CO2}\ CO_2 + \nu_{H2O}\ H_2O \rightarrow \nu_{S0}\ S^0 + \nu_{SO4}\ SO_4^{2-} + \nu_{orgC}\ CH_2O + \nu_{H+}\ H^+}$	$\mathbf{S_2O_3^{2-} + \nu_{O2}\ O_2 + \nu_{CO2}\ CO_2 + \nu_{H2O}\ H_2O \rightarrow \nu_{S0}\ S^0 + \nu_{SO4}\ SO_4^{2-} + \nu_{orgC}\ CH_2O + \nu_{H+}\ H^+}$
	$\nu_{O2} = y\ (2–1.5x)$	$\nu_{O2} = y\ (2–1.5x)$
	$\nu_{CO2} = (1–y)\ (2–1.5x)$	$\nu_{CO2} = (1–y)\ (2–1.5x)$
	$\nu_{H2O} = (1–y)\ (2–2.5x) – y\ x$	$\nu_{H2O} = y\ (1–x) + (1–y)\ (3–2.5x)$
	$\nu_{S0} = x$	$\nu_{S0} = x$
	$\nu_{SO4} = (1–x)$	$\nu_{SO4} = (2–x)$
	$\nu_{orgC} = (1–y)\ (2–1.5x)$	$\nu_{orgC} = (1–y)\ (2–1.5x)$
	$\nu_{H+} = (2–2x)$	$\nu_{H+} = (2–2x)$

[1]Equation for CO_2 fixation coupled to electron carrier oxidation (Eqs. 18 and 24) is the same for all sulfur oxidation processes.

Table S1B: Generalized mass-balanced equations for anaerobic oxidation of reduced sulfur species coupled to DNRA and CO_2 fixation.

	$\sum H_2S$ oxidation	$S_2O_3^{2-}$ oxidation
Energy-generating reactions[1]	$H_2S + 0.25\ NO_3^- + 0.5\ H^+ \rightarrow S^0 + 0.25\ NH_4^+ + 0.75\ H_2O$	$S_2O_3^{2-} + 0.25\ NO_3^- + 1.5\ H^+ \rightarrow S^0 + SO_4^{2-} + 0.25\ NH_4^+ + 0.25\ H_2O$
	$H_2S + NO_3^- + H_2O \rightarrow SO_4^{2-} + NH_4^+$	$S_2O_3^{2-} + NO_3^- + 2\ H_2O \rightarrow 2\ SO_4^{2-} + NH_4^+$
	$\mathbf{H_2S + (1–0.75\mathit{x})\ NO_3^- + (1–1.75\mathit{x})\ H_2O + 0.5\mathit{x}\ H^+ \rightarrow \mathit{x}\ S^0 + (1–\mathit{x})\ SO_4^{2-} + (1–0.75\mathit{x})\ NH_4^+}$	$\mathbf{S_2O_3^{2-} + (1–0.75\mathit{x})\ NO_3^- + (2–2.25\ \mathit{x})\ H_2O + 1.5\mathit{x}\ H^+ \rightarrow \mathit{x}\ S^0 + (2–\mathit{x})\ SO_4^{2-} + (1–0.75\mathit{x})\ NH_4^+}$
Generalized coupled reaction	$\mathbf{H_2S + \mathit{v_{NO3}}\ NO_3^- + \mathit{v_{CO2}}\ CO_2 + \mathit{v_{H2O}}\ H_2O \rightarrow \mathit{v_{S0}}\ S^0 + \mathit{v_{SO4}}\ SO_4^{2-} + \mathit{v_{NH4+}} NH_4^+ + \mathit{v_{orgC}}\ CH_2O + \mathit{v_{H+}}\ H^+}$	$\mathbf{S_2O_3^{2-} + \mathit{v_{NO3}}\ NO_3^- + \mathit{v_{CO2}}\ CO_2 + \mathit{v_{H2O}}\ H_2O \rightarrow \mathit{v_{S0}}\ S^0 + \mathit{v_{SO4}}\ SO_4^{2-} + \mathit{v_{NH4+}}\ NH_4^+ + \mathit{v_{orgC}}\ CH_2O + \mathit{v_{H+}}\ H^+}$
	$v_{NO3} = y\ (1–0.75x)$	$v_{NO3} = y\ (1–0.75x)$
	$v_{CO2} = (1–y)\ (2–1.5x)$	$v_{CO2} = (1–y)\ (2–1.5x)$
	$v_{H2O} = y\ (1–1.75x) + (1–y)\ (2–2.5x)$	$v_{H2O} = y\ (2–2.25x) + (1–y)\ (3–2.5x)$
	$v_{S0} = x$	$v_{S0} = x$
	$v_{SO4} = (1–x)$	$v_{SO4} = (2–x)$
	$v_{\mathrm{NH4}} = y\ (1–0.75x)$	$v_{\mathrm{NH4}} = y\ (1–0.75x)$
	$v_{orgC} = (1–y)\ (2–1.5x)$	$v_{orgC} = (1–y)\ (2–1.5x)$
	$v_{H+} = (1–y)\ (2–2x) – y\ 0.5x$	$v_{H+} = (1–y)\ (2–2x) – y\ 1.5x$

[1] The generalized energy conserving reactions are the same as in Table S1A.

Table S1C: Generalized mass-balanced equations for anaerobic oxidation of reduced sulfur species coupled to denitrification and CO_2 fixation.

	$\sum H_2S$ oxidation	$S_2O_3^{2-}$ oxidation
Energy-generating reactions[1]	$H_2S + 0.4\ NO_3^- \rightarrow S^0 + 0.2\ N_2 + 1.2\ H_2O + 0.6\ H^+$	$S_2O_3^{2-} + 0.4\ NO_3^- + 0.4\ H^+ \rightarrow S^0 + SO_4^{2-} + 0.2\ N_2 + 0.2\ H_2O$
	$H_2S + 1.6\ NO_3^- \rightarrow SO_4^{2-} + 0.8\ N_2 + 0.8\ H_2O + 0.4\ H^+$	$S_2O_3^{2-} + 1.6\ NO_3^- + 0.2\ H_2O \rightarrow 2\ SO_4^{2-} + 0.8\ N_2 + 0.4\ H^+$
	$\mathbf{H_2S + (1.6–1.2}x\mathbf{)\ NO_3^- + (- 0.8– 0.4}x\mathbf{)\ H_2O \rightarrow} x\ \mathbf{S^0 + (1–}x\mathbf{)\ SO_4^{2-} + (0.8–0.6}x\mathbf{)\ N_2 + (0.4 + 0.2}x\mathbf{)\ H^+}$	$\mathbf{S_2O_3^{2-} + (1.6–1.2}x\mathbf{)\ NO_3^- + (0.2– 0.4}x\mathbf{)\ H_2O \rightarrow} x\ \mathbf{S^0 + (2–}x\mathbf{)\ SO_4^{2-} + (0.8–0.6}x\mathbf{)\ N_2 + (0.4–0.8}x\mathbf{)\ H^+}$
Generalized coupled reaction	$\mathbf{H_2S} + v_{NO3}\ \mathbf{NO_3^-} + v_{CO2}\ \mathbf{CO_2} + v_{H2O}\ \mathbf{H_2O} \rightarrow v_{S0}\ \mathbf{S^0} + v_{SO4}\ \mathbf{SO_4^{2-}} + v_{N2}\ \mathbf{N_2} + v_{orgC}\ \mathbf{CH_2O} + v_{H+}\ \mathbf{H^+}$	$\mathbf{S_2O_3^{2-}} + v_{NO3}\ \mathbf{NO_3^-} + v_{CO2}\ \mathbf{CO_2} + v_{H2O}\ \mathbf{H_2O} \rightarrow v_{S0}\ \mathbf{S^0} + v_{SO4}\ \mathbf{SO_4^{2-}} + v_{N2}\ \mathbf{N_2} + v_{orgC}\ \mathbf{CH_2O} + v_{H+}\ \mathbf{H^+}$
	$v_{NO3} = y\ (1.6–1.2x)$	$v_{NO3} = y\ (1.6–1.2x)$
	$v_{CO2} = (1–y)\ (2–1.5x)$	$v_{CO2} = (1–y)\ (2–1.5x)$
	$v_{H2O} = y\ (–0.8–0.4x) + (1–y)\ (2–2.5x)$	$v_{H2O} = y\ (0.2– 0.4x) + (1–y)\ (3–2.5x)$
	$v_{S0} = x$	$v_{S0} = x$
	$v_{SO4} = (1–x)$	$v_{SO4} = (2–x)$
	$v_{N2} = y\ (0.8–0.6x)$	$v_{N2} = y\ (0.8–0.6x)$
	$v_{orgC} = (1–y)\ (2–1.5x)$	$v_{orgC} = (1–y)\ (2–1.5x)$
	$v_{H+} = y\ (0.4+0.2x) + (1–y)\ (2–2x)$	$v_{H+} = y\ (0.4–0.8x) + (1–y)\ (2–2x)$

[1] The generalized energy conserving reactions are the same as in Table S1A.

Table S1D: Generalized mass-balanced equations for anaerobic oxidation of reduced sulfur species coupled to nitrite reduction and CO_2 fixation.

	$\sum H_2S$ oxidation	$S_2O_3^{2-}$ oxidation
Energy-generating reactions[1]	$H_2S + 0.67\ NO_2^- + 0.67\ H^+ \rightarrow S^0 + 0.33\ N_2 + 1.33\ H_2O$	$S_2O_3^{2-} + 0.67\ NO_2^- + 0.67\ H^+ \rightarrow S^0 + SO_4^{2-} + 0.33\ N_2 + 0.33\ H_2O$
	$H_2S + 2.67\ NO_2^- + 0.67\ H^+ \rightarrow SO_4^{2-} + 1.33\ N_2 + 1.33\ H_2O$	$S_2O_3^{2-} + 2.67\ NO_2^- + 0.67\ H^+ \rightarrow 2\ SO_4^{2-} + 1.33\ N_2 + 0.33\ H_2O$
	$\mathbf{H_2S + (2.67{-}2\mathit{x})\ NO_2^- + 0.67\ H^+ \rightarrow \mathit{x}\ S^0 + (1{-}\mathit{x})\ SO_4^{2-} + (1.33{-}1\mathit{x})\ N_2 + 1.33\ H_2O}$	$\mathbf{S_2O_3^{2-} + (0.67 + 2\mathit{x})\ NO_2^- + ({-}0.33)\ H_2O \rightarrow \mathit{x}\ S^0 + (2{-}\mathit{x})\ SO_4^{2-} + (0.33{-}\mathit{x})\ N_2 + ({-}0.67)\ H^+}$
Generalized coupled reaction	$\mathbf{H_2S + \mathit{v_{NO2}}\ NO_2^- + \mathit{v_{CO2}}\ CO_2 + \mathit{v_{H2O}}\ H_2O \rightarrow \mathit{v_{S0}}\ S^0 + \mathit{v_{SO4}}\ SO_4^{2-} + \mathit{v_{N2}}\ N_2 + \mathit{v_{orgC}}\ CH_2O + \mathit{v_{H+}}\ H^+}$	$\mathbf{S_2O_3^{2-} + \mathit{v_{NO2}}\ NO_2^- + \mathit{v_{CO2}}\ CO_2 + \mathit{v_{H2O}}\ H_2O \rightarrow \mathit{v_{S0}}\ S^0 + \mathit{v_{SO4}}\ SO_4^{2-} + \mathit{v_{N2}}\ N_2 + \mathit{v_{orgC}}\ CH_2O + \mathit{v_{H+}}\ H^+}$
	$v_{NO2} = y\ (2.67{-}2x)$	$v_{NO2} = y\ (0.67{+}2x)$
	$v_{CO2} = (1{-}y)\ (2{-}1.5x)$	$v_{CO2} = (1{-}y)\ (2{-}1.5x)$
	$v_{H2O} = (1{-}y)\ (2{-}2.5x) - 1.33\ y$	$v_{H2O} = -0.33\ y + (1{-}y)\ (3{-}2.5x)$
	$v_{S0} = x$	$v_{S0} = x$
	$v_{SO4} = (1{-}x)$	$v_{SO4} = (1{-}x)$
	$v_{N2} = y\ (1.33{-}1x)$	$v_{N2} = y\ (0.33{-}x)$
	$v_{orgC} = (1{-}y)\ (2{-}1.5x)$	$v_{orgC} = (1{-}y)\ (2{-}1.5x)$
	$v_{H+} = (1{-}y)\ (2{-}2x) - 0.67\ y$	$v_{H+} = -0.67y + (1{-}y)\ (2{-}2x)$

[1] The generalized energy conserving reactions are the same as in Table S1A.

Table S1E: Generalized mass-balanced equations for anaerobic oxidation of reduced sulfur species coupled to N_2O reduction and CO_2 fixation.

	$\sum H_2S$ oxidation	$S_2O_3^{2-}$ oxidation
Energy-generating reactions[1]	$H_2S + N_2O \rightarrow S^0 + N_2 + H_2O$	$S_2O_3^{2-} + N_2O \rightarrow S^0 + SO_4^{2-} + N_2$
	$H_2S + 4\ N_2O \rightarrow SO_4^{2-} + 4\ N_2 + 2\ H^+$	$S_2O_3^{2-} + 4\ N_2O + H_2O \rightarrow 2\ SO_4^{2-} + 4\ N_2 + 2\ H^+$
	$\mathbf{H_2S + (4–3\mathit{x})\ N_2O \rightarrow \mathit{x}\ S^0 + (1–\mathit{x})\ SO_4^{2-} + (4–3\mathit{x})\ N_2 + \mathit{x}\ H_2O + (2–2\ \mathit{x})\ H^+}$	$\mathbf{S_2O_3^{2-} + (4–3\mathit{x})\ N_2O + (1–\mathit{x})\ H_2O \rightarrow \mathit{x}\ S^0 + (2–\mathit{x})\ SO_4^{2-} + (4–3\mathit{x})\ N_2 + (2–2\mathit{x})\ H^+}$
Generalized coupled reaction	$\mathbf{H_2S + \nu_{N2O}\ N_2O + \nu_{CO2}\ CO_2 + \nu_{H2O}\ H_2O \rightarrow \nu_{S0}\ S^0 + \nu_{SO4}\ SO_4^{2-} + \nu_{N2}\ N_2 + \nu_{orgC}\ CH_2O + \nu_{H+}\ H^+}$	$\mathbf{S_2O_3^{2-} + \nu_{N2O}\ N_2O + \nu_{CO2}\ CO_2 + \nu_{H2O}\ H_2O \rightarrow \nu_{S0}\ S^0 + \nu_{SO4}\ SO_4^{2-} + \nu_{N2}\ N_2 + \nu_{orgC}\ CH_2O + \nu_{H+}\ H^+}$
	$\nu_{N2O} = y\ (4–3x)$	$\nu_{N2O} = y\ (4–3x)$
	$\nu_{CO2} = (1–y)\ (2–1.5x)$	$\nu_{CO2} = (1–y)\ (2–1.5x)$
	$\nu_{H2O} = (1–y)\ (2–2.5x) – yx$	$\nu_{H2O} = y\ (1–x) + (1–y)\ (3–2.5x)$
	$\nu_{S0} = x$	$\nu_{S0} = x$
	$\nu_{SO4} = (1–x)$	$\nu_{SO4} = (2–x)$
	$\nu_{N2} = y\ (4–3x)$	$\nu_{N2} = y\ (3–2x)$
	$\nu_{orgC} = (1–y)\ (2–1.5x)$	$\nu_{orgC} = (1–y)\ (2–1.5x)$
	$\nu_{H+} = (2–2x)$	$\nu_{H+} = (2–2x)$

[1]The generalized energy conserving reactions are the same as in Table S1A.

Table S2: Stoichiometries of autotrophic sulfur oxidation calculated for specific SOB strains based on experimental data available in the literature.

		x	y	Reference(s)
	PART A: Aerobic thiosulfate oxidation			
Thiobacillus denitrificans	$S_2O_3^{2-} + 1.42\ O_2 + 0.58\ CO_2 + 1.58\ H_2O \rightarrow 2\ SO_4^{2-} + 0.58\ CH_2O + 2\ H^+$	0	0.710	(Justin and Kelly, 1978)
Thiobacillus denitrificans	$S_2O_3^{2-} + 1.48\ O_2 + 0.52\ CO_2 + 1.52\ H_2O \rightarrow 2\ SO_4^{2-} + 0.52\ CH_2O + 2\ H^+$	0	0.740	(Hoor, 1981)
Thiothrix CT3	$S_2O_3^{2-} + 1.48\ O_2 + 0.52\ CO_2 + 1.52\ H_2O \rightarrow 2\ SO_4^{2-} + 0.52\ CH_2O + 2\ H^+$	0	0.738	(Rossetti, 2003)
Beggiatoa str. D-402	$S_2O_3^{2-} + 1.52\ O_2 + 0.48\ CO_2 + 1.48\ H_2O \rightarrow 2\ SO_4^{2-} + 0.48\ CH_2O + 2\ H^+$	0	0.761	(Grabovich et al., 2001)
Thermothrix thiopara	$S_2O_3^{2-} + 1.57\ O_2 + 0.44\ CO_2 + 1.44\ H_2O \rightarrow 2\ SO_4^{2-} + 0.44\ CH_2O + 2\ H^+$	0	0.783	(Mason et al., 1987)
Thermithiobacillus tepidarius	$S_2O_3^{2-} + 1.61\ O_2 + 0.39\ CO_2 + 1.39\ H_2O \rightarrow 2\ SO_4^{2-} + 0.39\ CH_2O + 2\ H^+$	0	0.804	(Wood and Kelly, 1986)
Thioalkalivibrio versutus	$S_2O_3^{2-} + 1.69\ O_2 + 0.31\ CO_2 + 1.31\ H_2O \rightarrow 2\ SO_4^{2-} + 0.31\ CH_2O + 2\ H^+$	0	0.844	(Sorokin et al., 2001)
Acidithiobacillus ferrooxidans	$S_2O_3^{2-} + 1.70\ O_2 + 0.30\ CO_2 + 1.30\ H_2O \rightarrow 2\ SO_4^{2-} + 0.30\ CH_2O + 2\ H^+$	0	0.848	(Eccleston and Kelly, 1978)
Paracoccus versutus	$S_2O_3^{2-} + 1.71\ O_2 + 0.29\ CO_2 + 1.29\ H_2O \rightarrow 2\ SO_4^{2-} + 0.29\ CH_2O + 2\ H^+$	0	0.853	(Mason et al., 1987)
Halothiobacillus neapolitanus	$S_2O_3^{2-} + 1.72\ O_2 + 0.28\ CO_2 + 1.28\ H_2O \rightarrow 2\ SO_4^{2-} + 0.28\ CH_2O + 2\ H^+$	0	0.859	(Hempfling and Vishniac, 1967; Kelly, 1982)
Thiomicrospira thioparus	$S_2O_3^{2-} + 1.73\ O_2 + 0.27\ CO_2 + 1.27\ H_2O \rightarrow 2\ SO_4^{2-} + 0.27\ CH_2O + 2\ H^+$	0	0.863	(Kelly, 1982)
Sulfurimonas denitrificans	$S_2O_3^{2-} + 1.67\ O_2 + 0.33\ CO_2 + 1.33\ H_2O \rightarrow 2\ SO_4^{2-} + 0.33\ CH_2O + 2\ H^+$	0	0.833	(Hoor, 1981)
Acidithiobacillus thiooxidans	$S_2O_3^{2-} + 1.75\ O_2 + 0.25\ CO_2 + 1.25\ H_2O \rightarrow 2\ SO_4^{2-} + 0.25\ CH_2O + 2\ H^+$	0	0.874	(Mason et al., 1987)
Halothiobacillus halophilus	$S_2O_3^{2-} + 1.75\ O_2 + 0.25\ CO_2 + 1.25\ H_2O \rightarrow 2\ SO_4^{2-} + 0.25\ CH_2O + 2\ H^+$	0	0.875	(Wood and Kelly, 1991)
Thiothrix ramosa	$S_2O_3^{2-} + 1.75\ O_2 + 0.25\ CO_2 + 1.25\ H_2O \rightarrow 2\ SO_4^{2-} + 0.25\ CH_2O + 2\ H^+$	0	0.875	(Odintsova et al., 1993)
Thioalkalispira microaerophila	$S_2O_3^{2-} + 1.77\ O_2 + 0.23\ CO_2 + 1.23\ H_2O \rightarrow 2\ SO_4^{2-} + 0.23\ CH_2O + 2\ H^+$	0	0.884	(Sorokin, 2002)
Thioalkalimicrobium aerophilum	$S_2O_3^{2-} + 1.78\ O_2 + 0.22\ CO_2 + 1.22\ H_2O \rightarrow 2\ SO_4^{2-} + 0.22\ CH_2O + 2\ H^+$	0	0.891	(Sorokin et al., 2001)
Thiomicrospira halophila	$S_2O_3^{2-} + 1.78\ O_2 + 0.22\ CO_2 + 1.22\ H_2O \rightarrow 2\ SO_4^{2-} + 0.22\ CH_2O + 2\ H^+$	0	0.891	(Sorokin et al., 2006)
Thioalkalibacter halophilus	$S_2O_3^{2-} + 1.78\ O_2 + 0.22\ CO_2 + 1.22\ H_2O \rightarrow 2\ SO_4^{2-} + 0.22\ CH_2O + 2\ H^+$	0	0.891	(Banciu et al., 2008)
Thiobacillus thioparus E6	$S_2O_3^{2-} + 1.80\ O_2 + 0.20\ CO_2 + 1.20\ H_2O \rightarrow 2\ SO_4^{2-} + 0.20\ CH_2O + 2\ H^+$	0	0.898	(Smith and Kelly, 1988)
Thioclava pacifica	$S_2O_3^{2-} + 1.83\ O_2 + 0.17\ CO_2 + 1.17\ H_2O \rightarrow 2\ SO_4^{2-} + 0.17\ CH_2O + 2\ H^+$	0	0.913	(Sorokin et al., 2005)
Thioalkalimicrobium sibericum	$S_2O_3^{2-} + 1.83\ O_2 + 0.17\ CO_2 + 1.17\ H_2O \rightarrow 2\ SO_4^{2-} + 0.17\ CH_2O + 2\ H^+$	0	0.916	(Sorokin et al., 2001)
Thiomicrospira sp. Strain L-12	$S_2O_3^{2-} + 1.57\ O_2 + 0.13\ CO_2 + 0.93\ H_2O \rightarrow 1.8\ SO_4^{2-} + 0.2\ S^0 + 0.13\ CH_2O + 1.6\ H^+$	0.2	0.921	(Ruby and Jannasch, 1982)

	PART B: Thiosulfate oxidation coupled to denitrification			
Thiobacillus denitrificans	$S_2O_3^{2-} + 1.24\ NO_3^- + 0.45\ CO_2 + 0.83\ H_2O \rightarrow 2\ SO_4^{2-} + 0.62\ N_2 + 0.45\ CH_2O + 0.76\ H^+$	0	0.775	(Justin and Kelly, 1978)
Thiobacillus denitrificans	$S_2O_3^{2-} + 1.30\ NO_3^- + 0.38\ CO_2 + 0.73\ H_2O \rightarrow 2\ SO_4^{2-} + 0.65\ N_2 + 0.38\ CH_2O + 0.70\ H^+$	0	0.812	(Hoor, 1981)
Sulfurimonas hongkongensis	$S_2O_3^{2-} + 1.36\ NO_3^- + 0.30\ CO_2 + 0.61\ H_2O \rightarrow 2\ SO_4^{2-} + 0.68\ N_2 + 0.30\ CH_2O + 0.64\ H^+$	0	0.852	(Cai et al., 2014)
Sulfurimonas gotlandica	$S_2O_3^{2-} + 1.40\ NO_3^- + 0.25\ CO_2 + 0.55\ H_2O \rightarrow 2\ SO_4^{2-} + 0.70\ N_2 + 0.25\ CH_2O + 0.60\ H^+$	0	0.875	(Bruckner et al., 2013)
Sulfurimonas gotlandica-like Epsilonproteobacterium (environmental sample)	$S_2O_3^{2-} + 1.42\ NO_3^- + 0.23\ CO_2 + 0.52\ H_2O \rightarrow 2\ SO_4^{2-} + 0.71\ N_2 + 0.23\ CH_2O + 0.58\ H^+$	0	0.887	(Brettar et al., 2006)
Sulfurimonas denitrificans	$S_2O_3^{2-} + 1.44\ NO_3^- + 0.20\ CO_2 + 0.47\ H_2O \rightarrow 2\ SO_4^{2-} + 0.72\ N_2 + 0.20\ CH_2O + 0.56\ H^+$	0	0.902	(Hoor, 1981)
	PART C: Aerobic sulfide oxidation			
Riftia pachyptila symbionts	$H_2S + 1.12\ S^0 + 0.75\ O_2 + 2.3\ CO_2 + 3.42\ H_2O \rightarrow 2.12\ SO_4^{2-} + 2.3\ CH_2O + 4.24\ H^+$	-1.12	0.375	(Girguis et al., 2002)
Solemya reidi symbionts	$H_2S + 0.75\ O_2 + 1\ CO_2 + 0.83\ H_2O \rightarrow 0.83\ SO_4^{2-} + 0.17\ S^0 + 1\ CH_2O + 1.67\ H^+$	0.17	0.429	(Anderson et al., 1987)
Riftia pachyptila symbionts	$H_2S + 0.75\ O_2 + 0.75\ CO_2 + 0.42\ H_2O \rightarrow 0.67\ SO_4^{2-} + 0.33\ S^0 + 0.75\ CH_2O + 1.33\ H^+$	0.33	0.500	(Girguis and Childress, 2006)
Beggiatoa str. MS-81-1c	$H_2S + 1.23\ O_2 + 0.69\ CO_2 + 0.64\ H_2O \rightarrow 0.95\ SO_4^{2} + 0.05\ S^{0\ -} + 0.69\ CH_2O + 1.9\ H^+$	0.05	0.641	(Hagen and Nelson, 1997)
Ridgeia piscesae symbionts	$H_2S + 1.43\ O_2 + 0.57\ CO_2 + 0.57\ H_2O \rightarrow SO_4^{2-} + 0.57\ CH_2O + 2\ H^+$	0	0.714	(Nyholm et al., 2008)
Thermithiobacillus tepidarius	$H_2S + 1.62\ O_2 + 0.38\ CO_2 + 0.38\ H_2O \rightarrow SO_4^{2-} + 0.38\ CH_2O + 2\ H^+$	0	0.808	(Wood and Kelly, 1986)
Beggiatoa str. MS-81-6	$H_2S + 1.65\ O_2 + 0.35\ CO_2 + 0.35\ H_2O \rightarrow SO_4^{2-} + 0.35\ CH_2O + 2\ H^+$	0	0.825	(Hagen and Nelson, 1997)
Halothiobacillus neapolitanus	$H_2S + 1.72\ O_2 + 0.28\ CO_2 + 0.28\ H_2O \rightarrow SO_4^{2-} + 0.28\ CH_2O + 2\ H^+$	0	0.859	(Kelly, 1982)

8. References

Anderson, A. E., Childress, J. J., and Favuzzi, J. A. (1987). Net uptake of CO_2 Driven by Sulphide and Thiosulphate Oxidation in the Bacterial Symbiont-Containing Clam *Solemya Reidi. J. Exp. Biol.* 133, 1–31.

Banciu, H. L., Sorokin, D. Y., Tourova, T. P., Galinski, E. A., Muntyan, M. S., Kuenen, J. G., and Muyzer, G. (2008). Influence of salts and pH on growth and activity of a novel facultatively alkaliphilic, extremely salt-tolerant, obligately chemolithoautotrophic sufur-oxidizing Gammaproteobacterium *Thioalkalibacter halophilus* gen. nov., sp. nov. from South-Western Siber. *Extremophiles* 12, 391–404. doi:10.1007/s00792-008-0142-1.

Brettar, I., Labrenz, M., Flavier, S., Bötel, J., Kuosa, H., Christen, R., and Höfle, M. G. (2006). Identification of a *Thiomicrospira denitrificans*-like epsilonproteobacterium as a catalyst for autotrophic denitrification in the central Baltic Sea. *Appl. Environ. Microbiol.* 72, 1364–72. doi:10.1128/AEM.72.2.1364-1372.2006.

Bruckner, C. G., Mammitzsch, K., Jost, G., Wendt, J., Labrenz, M., and Jürgens, K. (2013). Chemolithoautotrophic denitrification of epsilonproteobacteria in marine pelagic redox gradients. *Environ. Microbiol.* 15, 1505–13. doi:10.1111/j.1462-2920.2012.02880.x.

Cai, L., Shao, M.-F., and Zhang, T. (2014). Non-contiguous finished genome sequence and description of *Sulfurimonas hongkongensis* sp. nov., a strictly anaerobic denitrifying, hydrogen- and sulfur-oxidizing chemolithoautotroph isolated from marine sediment. *Stand. Genomic Sci.* 9. doi:10.4056/494.

Eccleston, M., and Kelly, D. P. (1978). Oxidation kinetics and chemostat growth kinetics of *Thiobacillus ferrooxidans* on tetrathionate and thiosulfate. *J. Bacteriol.* 134, 718–27.

Fukui, M., Teske, A., Aßmus, B., Muyzer, G., and Widdel, F. (1999). Physiology, phylogenetic relationships, and ecology of filamentous sulfate-reducing bacteria (genus *Desulfonema*). *Arch. Microbiol.* 172, 193–203. doi:10.1007/s002030050760.

Girguis, P. R., and Childress, J. J. (2006). Metabolite uptake, stoichiometry and chemoautotrophic function of the hydrothermal vent tubeworm *Riftia pachyptila*: responses to environmental variations in substrate concentrations and temperature. *J. Exp. Biol.* 209, 3516–28. doi:10.1242/jeb.02404.

Girguis, P. R., Childress, J. J., Freytag, J. K., Klose, K., and Stuber, R. (2002). Effects of metabolite uptake on proton-equivalent elimination by two species of deep-sea vestimentiferan tubeworm, *Riftia pachyptila* and *Lamellibrachia* cf *luymesi*: proton elimination is a necessary adaptation to sulfide-oxidizing chemoautotrophic symbiont. *J. Exp. Biol.* 205, 3055–3066.

Grabovich, M. Y., Patritskaya, V. Y., Muntyan, M. S., and Dubinina, G. A. (2001). Lithoautotrophic growth of the freshwater strain *Beggiatoa* D-402 and energy conservation in a homogeneous culture under microoxic conditions. *FEMS Microbiol. Lett.* 204, 341–5.

Hagen, K. D., and Nelson, D. C. (1997). Use of Reduced Sulfur Compounds by *Beggiatoa* spp.: Enzymology and physiology of marine and freshwater strains in homogeneous and gradient cultures. *Appl. Environ. Microbiol.* 63, 3957–3964.

Hempfling, W. P., and Vishniac, W. (1967). Yield coefficients of Thiobacillus *neapolitanus* in continuous culture. *J. Bacteriol.* 93, 874–8.

Hoor, A. T. (1981). Cell yield and bioenergetics of *Thiomicrospira denitrificans* compared with *Thiobacillus denitrificans*. *Antonie Van Leeuwenhoek* 47, 231–243. doi:10.1007/BF00403394.

Justin, P., and Kelly, D. P. (1978). Growth kinetics of *Thiobacillus denitrificans* in anaerobic and aerobic chemostat culture. *J. Gen. Microbiol.* 107, 123–130. doi:10.1099/00221287-107-1-123.

Kelly, D. P. (1982). Biochemistry of the chemolithotrophic oxidation of inorganic sulphur. *Philos. Trans. R. Soc. Lond. B. Biol. Sci.* 298, 499–528. doi:10.1098/rstb.1982.0094.

Mason, J., Kelly, D. P., and Wood, A. P. (1987). Chemolithotrophic and autotrophic growth of *Thermothrix thiopara* and some thiobacilli on thiosulphate and polythionates, and a reassessment of the growth yields of *Thx. thiopara* in chemostat culture. *Microbiology* 133, 1249–1256. doi:10.1099/00221287-133-5-1249.

Nyholm, S. V., Robidart, J., and Girguis, P. R. (2008). Coupling metabolite flux to transcriptomics: Insights into the molecular mechanisms underlying primary productivity by the hydrothermal vent tubeworm *Ridgeia piscesae*. *Biol. Bull.* 214, 255–265.

Odintsova, E. V., Wood, A. P., and Kelly, D. P. (1993). Chemolithoutotrophic growth of *Thiothrix ramosa. Arch. Microbiol.* 160, 152–157. doi:10.1007/BF00288718.

Otte, S., Kuenen, J., Nielsen, L., Paerl, H., Zopfi, J., Schulz, H., Teske, A., Strotmann, B., Gallardo, V., and Jorgensen, B. (1999). Nitrogen, carbon, and sulfur metabolism in natural *Thioploca* samples. *Appl. Environ. Microbiol.* 65, 3148–57.

Rossetti, S. (2003). Phylogenetic and physiological characterization of a heterotrophic, chemolithoautotrophic *Thiothrix* strain isolated from activated sludge. *Int. J. Syst. Evol. Microbiol.* 53, 1271–1276. doi:10.1099/ijs.0.02647-0.

Ruby, E. G., and Jannasch, H. W. (1982). Physiological characteristics of *Thiomicrospira* sp. Strain L-12 isolated from deep-sea hydrothermal vents. *J. Bacteriol.* 149, 161–165.

Simon, M., and Azam, F. (1989). Protein content and protein synthesis rates of planktonic marine bacteria. *Mar. Ecol. Prog. Ser.* 51, 201–213.

Smith, N. A., and Kelly, D. P. (1988). Mechanism of oxidation of dimethyl disulphide by *Thiobacillus thioparu*s strain E6. *Microbiology* 134, 3031–3039. doi:10.1099/00221287-134-11-3031.

Sorokin, D. Y. (2002). *Thioalkalispira microaerophila* gen. nov., sp. nov., a novel lithoautotrophic, sulfur-oxidizing bacterium from a soda lake. *Int. J. Syst. Evol. Microbiol.* 52, 2175–2182. doi:10.1099/ijs.0.02339-0.

Sorokin, D. Y., Lysenko, A. M., Mityushina, L. L., Tourova, T. P., Jones, B. E., Rainey, F. A., Robertson, L. A., and Kuenen, G. J. (2001). *Thioalkalimicrobium aerophilum* gen. nov., sp. nov. and *Thioalkalimicrobium sibericum* sp. nov., and *Thioalkalivibrio versutus* gen. nov., sp. nov., *Thioalkalivibrio nitratis* sp.nov., novel and *Thioalkalivibrio denitrificancs* sp. nov., novel obligately alkaliphilic and obligately chemolithoautotrophic sulfur-oxidizing bacteria from soda lakes. *Int. J. Syst. Evol. Microbiol.* 51, 565–80. doi:10.1099/00207713-51-2-565.

Sorokin, D. Y., Tourova, T. P., Kolganova, T. V, Spiridonova, E. M., Berg, I. A., and Muyzer, G. (2006). *Thiomicrospira halophila* sp. nov., a moderately halophilic, obligately chemolithoautotrophic, sulfur-

oxidizing bacterium from hypersaline lakes. *Int. J. Syst. Evol. Microbiol.* 56, 2375–80. doi:10.1099/ijs.0.64445-0.

Sorokin, D. Y., Tourova, T. P., Spiridonova, E. M., Rainey, F. A., and Muyzer, G. (2005). *Thioclava pacifica* gen. nov., sp. nov., a novel facultatively autotrophic, marine, sulfur-oxidizing bacterium from a near-shore sulfidic hydrothermal area. *Int. J. Syst. Evol. Microbiol.* 55, 1069–75. doi:10.1099/ijs.0.63415-0.

Teske, A., Jørgensen, B. B., and Gallardo, V. A. (2009). Filamentous bacteria inhabiting the sheaths of marine *Thioploca* spp. on the Chilean continental shelf. *FEMS Microbiol. Ecol.* 68, 164–72. doi:10.1111/j.1574-6941.2009.00659.x.

Wood, A. P., and Kelly, D. P. (1986). Chemolithotrophic metabolism of the newly-isolated moderately thermophilic, obligately autotrophic *Thiobacillus tepidarius*. *Arch. Microbiol.* 144, 71–77. doi:10.1007/BF00454959.

Wood, A. P., and Kelly, D. P. (1991). Isolation and characterisation of *Thiobacillus halophilus* sp. nov., a sulphur-oxidising autotrophic eubacterium from a Western Australian hypersaline lake. *Arch. Microbiol.* 156, 277–280. doi:10.1007/BF00262998.
